# Tumor-derived miR-20b-5p promotes lymphatic metastasis of esophageal squamous cell carcinoma by remodeling the tumor microenvironment

**DOI:** 10.1038/s41392-022-01242-1

**Published:** 2023-01-25

**Authors:** Zitong Zhao, Liyan Xue, Leilei Zheng, Liying Ma, Zhuo Li, Ning Lu, Qimin Zhan, Yongmei Song

**Affiliations:** 1 State Key Laboratory of Molecular Oncology, National Cancer Center/National Clinical Research Center for Cancer/Cancer Hospital, Chinese Academy of Medical Sciences and Peking Union Medical College, Beijing, China; 2 Department of Pathology, National Cancer Center/National Clinical Research Center for Cancer/Cancer Hospital, Chinese Academy of Medical Sciences and Peking Union Medical College, Beijing, China; 3 Laboratory of Molecular Oncology, Peking University Cancer Hospital, Beijing, China

**Keywords:** Gastrointestinal cancer, Metastasis, Cancer microenvironment, Non-coding RNAs

## Dear Editor,

Esophageal squamous cell carcinoma (ESCC) is one of the most common fatal malignancies worldwide and is especially common in East Asian regions, including China. Screening lymph node metastasis (LNM)-related biomarkers and elucidating the mechanism could provide promising therapeutic targets and help ESCC patients to select reasonable individual therapies. Recent studies have shown that extracellular vesicles (EVs) secreted by tumors contain miRNAs and that miRNAs in EVs can rebuild the tumor microenvironment (TME), thereby providing favorable conditions for tumor metastasis. However, whether specific EVs can be derived from ESCC cells and what affects EVs-mediated communication between ESCC cells and recipient cells in the process of LNM have not been investigated.

**Fig. 1:**
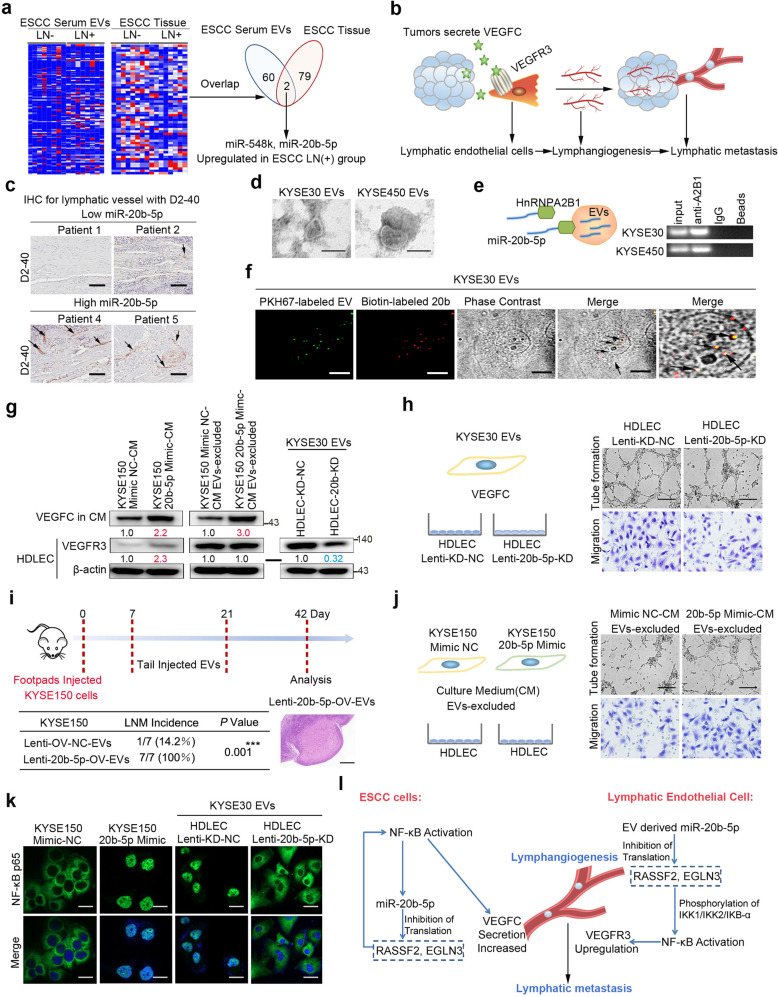
EV miR-20b-5p derived from ESCC cells induced lymphangiogenesis of lymphatic endothelial cells facilitate lymphatic metastasis by remodeling the tumor microenvironment. **a** Heat-map of small RNA sequencing in ESCC serum EVs (4 patients with LNM and 4 patients without LNM) and ESCC tissue (4 patients with LNM and 4 patients without LNM). **b** Schematic model of VEGFC/VEGFR3-mediated lymphangiogenesis and LNM. **c** Representative IHC staining of D2-40(lymphatic vessel) in ESCC patients with low and high miR-20b-5p level. Scale bar, 100 μm. **d** Electron microscopy images of EVs secreted by KYSE30 and KYSE450 cells. Scale bar, 100 nm. **e** RIP assay to assess the effect of hnRNPA2B1 on miR-20b-5p relative to IgG in KYSE30 and KYSE450 cells. Gel electrophoresis of the PCR products from the RIP assay. **f** Fluorescence observation of HDLECs at 24 h after incubation with PKH67-labeled (green) EVs carried biotin-miR-20b-5p (red) from KYSE30. Representative fluorescence images and phase-contrast images are shown. Scale bar, 30 μm. **g** Western blot analysis of VEGFR3 in HDLECs and VEGFC in culture medium (CM). **h** Representative images of HDLEC-Lenti-miR-20b-5p-KD or HDLEC-Lenti-KD-NC and cultured with EVs from KYSE30 cells and VEGFC. Matrigel tube formation assay (scale bar, 2 mm) and migration assay (Original magnification, ×100). **i** Volumes of popliteal lymph nodes and rates of lymphatic metastasis as determined by HE analysis. KYSE150 cells were injected into the foot pads of nude mice to establish the LNM model. Seven days after cell injection, the mice were randomly assigned to one of two groups receiving tail injections of EVs from either KYSE150-Lenti-OV-NC or KYSE150-Lenti-miR-20b-5p-OV cells. Lymphatic metastasis of representative tumors was determined by HE analysis. Scale bar, 200 μm. **j** Representative images of HDLECs cultured with CM (devoid of EVs) from KYSE150 miR-20b-5p-overexpressing cells and corresponding control cells. Matrigel tube formation assay (scale bar, 2 mm) and migration assay (original magnification, ×100). **k** Immunofluorescence analysis of NF-κB sublocalization (green) as determined by confocal microscopy. Scale bar, 30 μm. **l** Model of EV miR-20b-5p remodeling the tumor microenvironment by inducing lymphangiogenesis of LECs in ESCC LNM. The data are representative of three independent experiments. The error bars represent the SEM. ***P < 0.001, two-tailed unpaired Student’s t-test

To identify EV-riched miRNAs derived from tumor-mediating ESCC LNM, transcript sequencing was performed in serum EV and tissue from ESCC patients. The results revealed that miR-20b-5p and miR-548k were upregulated in both serum EV and tissue from ESCC patients with LNM (Fig. 1a). We have elucidated the function and mechanism of miR-548k in ESCC before,^1^ and found that miR-20b-5p promoted aggressive phenotype of ESCC cells (Supplementary Fig. S1). We next found that miR-20b-5p expression was significantly higher in ESCC patients with LNM in a larger sample size (Supplementary Fig. S2a). Tumor lymphangiogenesis is necessary for LNM and involves lymphatic endothelial cell (LEC) proliferation, migration, and lumen structure formation (Fig. 1b).^2^ The expression of miR-20b-5p in ESCC FFPE and serum EVs was positively correlated with the number and density of lymphatic vessels, VEGFC level in serum (Fig. 1c, Supplementary Fig. S2b–d, and Supplementary Tables S1, 2).

EVs were isolated by ultracentrifugation and verified by transmission electron microscopy, NanoSight analysis and Western blotting (Fig. 1d and Supplementary Fig. 3b–e). We next identified miR-20b-5p in the cell supernatant was mainly derived from EVs from ESCC cells (Supplementary Fig. 3f, g). Specific sequence (GGAG) has been identified as EV motifs, which can be specifically recognized by hnRNPA2B1, thereby regulating the selective entry of these miRNAs into EVs.^3^ We found two sequences that are similar to GGAG at the 3’ end of miR-20b-5p, and RIP assays showed that hnRNPA2B1 could bind to miR-20b-5p, then we found that hnRNPA2B1 carried miR-20b-5p into EVs (Fig. 1e and Supplementary Fig. 3h–l). EVs carrying miR-20b-5p derived from ESCC could be uptaken by human dermal lymphatic endothelial cells (HDLECs) (Fig. 1f).

VEGFC is the main lymphangiogenic factor that binds to the receptor VEGFR3, which is specifically expressed in LECs. The VEGFC/VEGFR3 pathway is regarded as the principal inducer of lymphangiogenesis.^2^ After overexpressing or knocking down miR-20b-5p in ESCC cells, we collected the cell supernatants and cocultured them together with HDLECs. Compared with that in the control group, the cell migration, tube formation and expression of VEGFR3 of HDLEC in the supernatants of cells with miR-20b-5p overexpression and knockdown was up- and downregulated, respectively (Fig. 1g and Supplementary Fig. S4a, b). We constructed KYSE150 cells with stable and high miR-20b-5p expression for animal experiments (Supplementary Fig. S5a). The subcutaneous tumorigenesis experiment showed that compared with control group tumors, the tumor formation ability of KYSE150 cells expressing miR-20b-5p at high levels was stronger, the density and number of lymphatic vessels in ESCC cells with high miR-20b-5p expression were higher, and the expression of VEGFC was higher (Supplementary Fig. S5b–e). The lung metastasis was higher in tumors comprised of KYSE150 cells with high miR-20b-5p expression than control group (Supplementary Fig. S5f). Injection of tumor cells into the nude mouse foot pad serves as an animal model for observing LNM. Compared with that in control group mice, the incidence of LNM was higher in nude mice with high miR-20b-5p expression (Supplementary Fig. S5g).

We speculated that EV-riched miR-20b-5p secreted by ESCC may alter the state of some key cells in the tumor microenvironment, such as LECs. To prove this conjecture, we constructed a stable HDLECs miR-20b-5p knockdown line and treated cells with EVs isolated from ESCC cells. The expression of VEGFR3 in EVs treated HDLECs with stable miR-20b-5p knockdown was downregulated, and the cell migration and tube formation abilities were weakened (Fig. 1g, h and Supplementary Fig. 6a-e). We overexpressed miR-20b-5p in HDLECs and treated the control and experimental cells with the supernatants of ESCC cells. The expression of VEGFR3 in HDLECs with high miR-20b-5p expression was upregulated, and the migration and tube formation of HDLECs were enhanced (Supplementary Fig. 6f). These indicated that EV miR-20b-5p could upregulate VEGFR3 expression in HDLECs. To verify the effect of miR-20b-5p in EVs on LNM in nude mice, we collected EVs from KYSE150 cells with stable high miR-20b-5p expression and control cells (Supplementary Fig. 6g). The incidence of LNM was higher in mice with high EV miR-20b-5p expression than the control group (Fig. 1i).

The supernatant devoid of EVs was then cocultured with HDLECs. Compared with that in the control group, the VEGFC in the supernatants of cells with miR-20b-5p overexpression and knockdown (devoid of EVs) was up- and downregulated, and the migration and tube formation abilities of the HDLECs were increased and decreased, respectively. However, the VEGFR3 expression of HDLEC was not significantly different between the two groups (Fig. 1g, j and Supplementary Fig. 7a-c). These results suggested that miR-20b-5p induced lymphangiogenesis by affecting the ESCC cell secretion of VEGFC. Meanwhile, miR-20b-5p in ESCC cells and EVs-riched miR-20b-5p derived from ESCC cells had an additive effect on the migration and tube formation abilities of the HDLECs (Supplementary Fig. 8).

To elucidate the mechanism by which miR-20b-5p regulated lymphangiogenesis induced by VEGFC/VEGFR3, we performed transcript sequencing analysis of ESCC cells and HDLECs treated with EVs (Supplementary Fig. 9a, b and Supplementary Tables S3, 4). The p65 transcription factor in the NF-κB signaling pathway has been reported to transactivate the expression of VEGFC and VEGFR3. We identified RASSF2 and EGLN3, which were involved in the NF-κB/p65 signaling pathway,^4,5^ as target genes of miR-20b-5p by dual-luciferase reporter assay, qPCR and Western blotting in ESCC cells and HDLECs with EVs from the supernatants of ESCC cells (Supplementary Fig. 9c–h).

We verified that p65 could indeed bind to the promoter region of miR-20b-5p by ChIP (Supplementary Fig. 10a, b). Treatment of the cells with the NF-κB activator upregulated miR-20b-5p, while the expression of miR-20b-5p was relatively decreased when cells were treated with the NF-κB inhibitor (Supplementary Fig. 10c). Compared with the control group, overexpression of miR-20b-5p and knockdown of RASSF2/EGLN3 increased the nuclear entry of p65 and the NF-κB transcriptional activity in ESCC cells/HDLECs (Fig. 1k and Supplementary Figs. 11–13). Knockdown of miR-20b-5p and overexpression RASSF2/EGLN3 decreased the nuclear entry of p65 and the NF-κB transcriptional activity in ESCC cells and HDLECs with stable miR-20b-5p knockdown were treated with EVs (Supplementary Figs. 11–13). These results suggested that miR-20b-5p promoted the entry of p65 into the nucleus. After p65 entered the nucleus, it acted as a transcription factor to promote the expression of miR-20b-5p, thereby forming a positive feedback loop.

To verify the molecular mechanism by which miR-20b-5p promoted the entry of p65 into the nucleus by downregulating RASSF2 and EGLN3, we overexpressed miR-20b-5p in ESCC cells. We found that while RASSF2 and EGLN3 expression was decreased, IKK1, IKK2, and p65 phosphorylation was upregulated, the total protein level of IKB-α was decreased, and VEGFC was significantly upregulated. We knocked down miR-20b-5p in ESCC cells and found that while RASSF2 and EGLN3 expression was increased, IKK1, IKK2, and p65 phosphorylation was downregulated, the total protein level of IKB-α was increased, and VEGFC was significantly downregulated (Supplementary Fig. 14). Together, these results suggested that the NF-κB signaling pathway was activated by miR-20b-5p and then transactivated VEGFC.

To investigate the function of the miR-20b-5p-RASSF2/EGLN3-NF-κB axis in ESCC, we overexpressed RASSF2/EGLN3 in ESCC cells already overexpressing miR-20b-5p, or coexpressed a miR-20b-5p inhibitor and siRNAs targeting EGLN3/RASSF2 in ESCC cells; the supernatants were collected and cocultured with HDLECs (Supplementary Fig. 15). The results showed that RASSF2/EGLN3 partially rescued the effect of miR-20b-5p on HDLEC migration and tube formation and on the malignant phenotype of ESCC cells (Supplementary Figs. 15, 16).

While the overexpression of miR-20b-5p in HDLECs decreased RASSF2 and EGLN3 expression, the phosphorylation levels of IKK1, IKK2 and p65 were upregulated, the total IKB-α protein content was decreased, and the expression of VEGFR3 was increased significantly (Supplementary Fig. 17). Compared with the control group cells, HDLECs with stable miR-20b-5p knockdown treated with EVs exhibited decreased phosphorylation levels of IKK1, IKK2 and p65, increased IKB-α total protein contents, and obviously decreased VEGFR3 expression (Supplementary Fig. 17). Using CM from ESCC cells treated with HDLECs, the co-overexpression of miR-20b-5p and EGLN3/RASSF2 in HDLECs partially rescued the effects of miR-20b-5p overexpression on the migration and tube formation of HDLECs (Supplementary Fig. 18). The knockdown of EGLN3/RASSF2 in HDLECs with stable miR-20b-5p knockdown partially rescued the effect of miR-20b-5p downregulation on the migration and tube formation abilities of HDLECs (Supplementary Fig. 18).

Our findings identified EVs-miR-20b-5p as a potential biomarker for predicting the LNM of ESCC. These findings indicated that ESCC-derived EV miR-20b-5p remodeled the tumor microenvironment by inducing lymphangiogenesis of LECs in ESCC LNM and provided novel insight into the mechanism underlying LNM in ESCC (Fig. 1l, Supplementary Fig. 19).

## Supplementary information

SUPPLEMENTAL MATERIAL-clean

Table S4

## Data Availability

The raw transcriptome data have been deposited to the Sequence Read Archive (SRA). SRA accession numbers: PRJNA858972.
