# Supplementary material for: Tumor-derived miR-20b-5p promotes lymphatic metastasis of esophageal squamous cell carcinoma by remodeling the tumor microenvironment
Source: Signal Transduct Target Ther. 2023 Jan 25;8:29. doi: 10.1038/s41392-022-01242-1 (PMC9873610; doi:10.1038/s41392-022-01242-1)
Supplement: Supplementary file 1 — SUPPLEMENTAL MATERIAL-clean [file 41392_2022_1242_MOESM1_ESM.docx]

**Supplemental Material for**

**Tumor-derived miR-20b-5p promotes lymphatic metastasis of esophageal squamous cell carcinoma by remodeling the tumor microenvironment**

**Zitong Zhao^1#^, Liyan Xue^2#^, Leilei Zheng^1#^, Liying Ma^1^, Zhuo Li^2^, Ning Lu^2*^, Qimin Zhan^1,3*^, Yongmei Song^1*^**

^1^State Key Laboratory of Molecular Oncology, National Cancer Center/National Clinical Research Center for Cancer/Cancer Hospital, Chinese Academy of Medical Sciences and Peking Union Medical College, Beijing, China

^2^Department of Pathology, National Cancer Center/National Clinical Research Center for Cancer/Cancer Hospital, Chinese Academy of Medical Sciences and Peking Union Medical College, Beijing, China

^3^Laboratory of Molecular Oncology, Peking University Cancer Hospital, Beijing, China

These authors contributed equally: Zitong Zhao, Liyan Xue, Leilei Zheng

**Correspondence to:**

Professor Yongmei Song, symlh2006@163.com, songym@cicams.ac.cn;

Professor Qimin Zhan, zhanqimin@bjmu.edu.cn;

Professor Ning Lu, lvning@cicams.ac.cn.

**This file includes:**

Materials and Methods

Figures. S1 to S19

Tables. S1 to S4

**Materials and Methods**

**Patients and specimen collection**

A total of 122 T1 ESCC FFPE tissues and matched serum samples were collected at the National Cancer Center/National Clinical Research Center for Cancer/Cancer Hospital, Chinese Academy of Medical Sciences and Peking Union Medical College.

**Cell culture**

KYSE150, KYSE140, YES2, KYSE410, KYSE510, KYSE30, KYSE450, and human dermal lymphatic endothelial cells (HDLECs) were cultured in RPMI 1640 medium supplemented with 10% FBS and antibiotics. The esophageal immortalized NE3 cells were kindly provided by Professor Enmin Li from Shantou University and cultured in a 1:1 mixture of EpiLife and dKSFM (Gibco). All cell lines were incubated in a humidified incubator at 37°C and 5% CO_2_.

**RNA extraction, RT-PCR and quantitative real-time PCR (qPCR)**

Total RNA was extracted from FFPE tissues after macrodissection with an RNAprep Pure FFPE Kit (TIANGEN, China), and miRNAs were extracted from FFPE tissues with the miRNAprep Pure FFPE Kit (TIANGEN, China). Total RNA was extracted from frozen fresh tissue and cell lines with TRIzol reagent (Invitrogen, USA), precipitated with chloroform, and cleaned up using the RNAExpress Total RNA Kit (NCM Biotech, China) which uses spin columns instead of ethanol precipitation to purify the RNA. cDNA was synthesized with Superscript II reverse transcriptase (Invitrogen, USA). qPCR was performed with a SYBR Premix Ex TaqTM II kit (TaKaRa, Japan). The qPCR conditions included 1 cycle at 95°C for 2 min, followed by 40 cycles of amplification at 95°C for 5 s and 60°C for 30 s. The length of qPCR amplicon was 80~300bp. The qPCR primers and used in this study are listed in Supplementary Table 1.

U6 was used as an internal reference for miRNAs in ESCC FFPE tissues. Considering the current lack of a recognized miRNA standard internal reference gene in serum, we used an external reference for more scientific and rigorous quantification. We utilized the long-chain noncoding RNA GM13008, which is expressed in only mice and not in humans, as an external reference (to prevent the interference of human endogenous genes). Full length of mouse lncRNA-GM13008 was synthesized using MEGAscript T7 Transcription Kit (Invitrogen, USA) according to the manufacturer's protocol. The miR-20b-5p levels in serum EV were normalized against the synthesized exogenous reference, GM13008.

**HDLEC tube formation assay**

Cells were grown on 100-mm plates in about 80% confluence and cultured with media free of serum for 24 h. Conditioned media were collected after centrifugation at 2000 rpm and stored at -80°C. HDLECs (2×10^5^) were suspended in a mixture of conditioned medium (500 μL) and DMEM (500 μL) supplemented with 10% FBS and seeded on a 24-well plate coated with 50% Matrigel (300 μL/well). Tube formation was observed after incubation for 3 h at 37°C. The number of tubular structures was counted in each field.

**Analysis of *in vivo* tumorigenicity**

Four-week-old male BALB/c nude mice were provided by Vital River for the *in vivo* tumorigenicity study. The subcutaneous tumor model was established by injecting 5×10^5^ KYSE150 cells on the top backs of the mice. Four weeks later, the mice were sacrificed, and the volume of the transplanted tumors were measured. The lung metastases model was established by injecting 1×10^6^ KYSE150 cells in the tail veins of nude mouse. Five weeks later, the mice were sacrificed, and the incidence of lung metastases were observed and counted. The LNM model was established by injecting 1×10^6^ KYSE150 cells in the food pad of nude mouse. Five weeks later, the mice were sacrificed, and the incidence of LNM were observed and counted.

**EV isolation, characterization and nanoparticle tracking analysis**

EVs were isolated by differential centrifugation or RiboTM Exosome Isolation Reagent. EVs were examined by JEM-1400 Plus transmission electron microscopy (JELO, Japan) using negative staining and quantified by a NanoSight NS300 instrument (Malvern Instruments Ltd. UK) equipped with NTA 3.0 analytical software (Malvern Instruments Ltd. UK).

***In vitro* EV transfer**

Cells were transfected with biotin-labeled miR-20b-5p. Equivalent numbers of cells were plated in EV-free medium and cultured for EV isolation. For EV labeling, PKH76 (Sigma, USA) was added to the EV suspension and incubated for 5 min. PKH76-labeled EVs were incubated with HDLECs for 24 h. The cells were fixed in 4% paraformaldehyde and incubated in 1% BSA to prevent nonspecific protein–protein interactions. The biotin-labeled miR-20b-5p in the fixed cells was labeled with a rabbit anti-biotin/RBITC secondary antibody (BIOSS, China). Images were acquired by laser-scanning confocal microscopy (Leica, Germany) and analyzed using Photoshop CS4 (Adobe, USA).

**Western blot analysis**

These assays were performed as previously described using the following antibodies: anti-RASSF2 at the dilution of 1:500, anti-EGLN3 at the dilution of 1:500, anti-VEGFC at the dilution of 1:500, anti-LaminB1 at the dilution of 1:1000, anti-HnRNPA2B1 at the dilution of 1:500 (Proteintech, China). Anti-β-actin at the dilution of 1:5000, anti-IKK1 at the dilution of 1:500, anti-IKK2 at the dilution of 1:500, anti-TSG101 at the dilution of 1:500 (Santa Cruz Biotechnology, USA). Anti-CD63 at the dilution of 1:500, anti-p-IκB-α at the dilution of 1:500, anti-IκB-α at the dilution of 1:500, anti-p-NF-κB p65 at the dilution of 1:500, anti--NF-κB p65 at the dilution of 1:500, and anti-VEGFR3 at the dilution of 1:500 (Cell Signaling Technology, USA). Anti-Flag at the dilution of 1:1000 (Sigma, USA).

**Plasmid construction**

The RASSF2/EGLN3 3’UTR (WT) or mutant (MUT) with a predicted miR-20b-5p responsive element was inserted downstream of the firefly luciferase gene in the pmirGLO plasmid. The miR-20b-5p promoter (-2000 bp ~ 0 bp) was inserted upstream of the firefly luciferase gene in the pGL3.0 basic vector. A RASSF2/EGLN3 overexpression plasmid was constructed with the GV230 vector. The pNF-κB-luc (NF-κB response element) plasmid and hnRNPA2B1(fused with flag tag) plasmid were purchased from PPL.

**Luciferase reporter assay**

The luciferase plasmid and internal control plasmid pRL-SV40 were co-transfected into cells for 48h. The luciferase assay was performed by using a dual-luciferase reporter assay system (Promega). Luciferase activity was normalized against an internal control to correct for the variations in transfection efficiency.

**Retroviral infection**

The lentiviruses for the miR-20b-5p overexpressiong and knockdown were purchased from GeneChem and used to infect cells according to the manufacturer’s protocol. Forty-eight hours after infection, cells were selected with 1 μg/mL puromycin in the culture medium and maintain the selection for 48 h. Then stable transfectants were appraised and cultured with 0.5 μg/mL puromycin in the culture medium.

**ChIP assay**

Chromatin immunoprecipitation (ChIP) assays were performed using a Pierce™ Magnetic ChIP Kit (Thermo Scientific™,USA). Immunoprecipitation was performed with anti- NF-κB p65 antibodies (Cell Signaling Technology, USA). Specific regions were quantified by qRT-PCR using the primers listed in Supplementary Table 1.

**RIP assay**

The EZ-Magna RIP Kit (Millipore, USA) was applied to conduct the RIP assay according to the manufacturer’s protocol. Immunoprecipitation was performed with an anti-HnRNPA2B1 antibody (Proteintech, China).

**Immunohistochemistry (IHC)**

IHC of tumor tissues was performed using PV-9000 and DAB chromogenic kit (ZSGB-BIO, China) according to the manufacturer’s protocol using the following antibodies: anti-VEGFC at the dilution of 1:200, anti-LYVE-1 at the dilution of 1:200, anti-hnRNPA2B1 at the dilution of 1:200 (Proteintech, China), and anti-D2-40 at the dilution of 1:50(ZSGB-BIO, China).

**ELISA assay**

VEGFC level in serum was detected using Human VEGF-C ELISA Kit (Proteintech, China) according to the manufacturer’s protocol.

**Statistical analyses**

Statistical analyses were performed using GraphPad 6.0 and SPSS 17.0 for Windows. Significance was considered *P*<0.05.


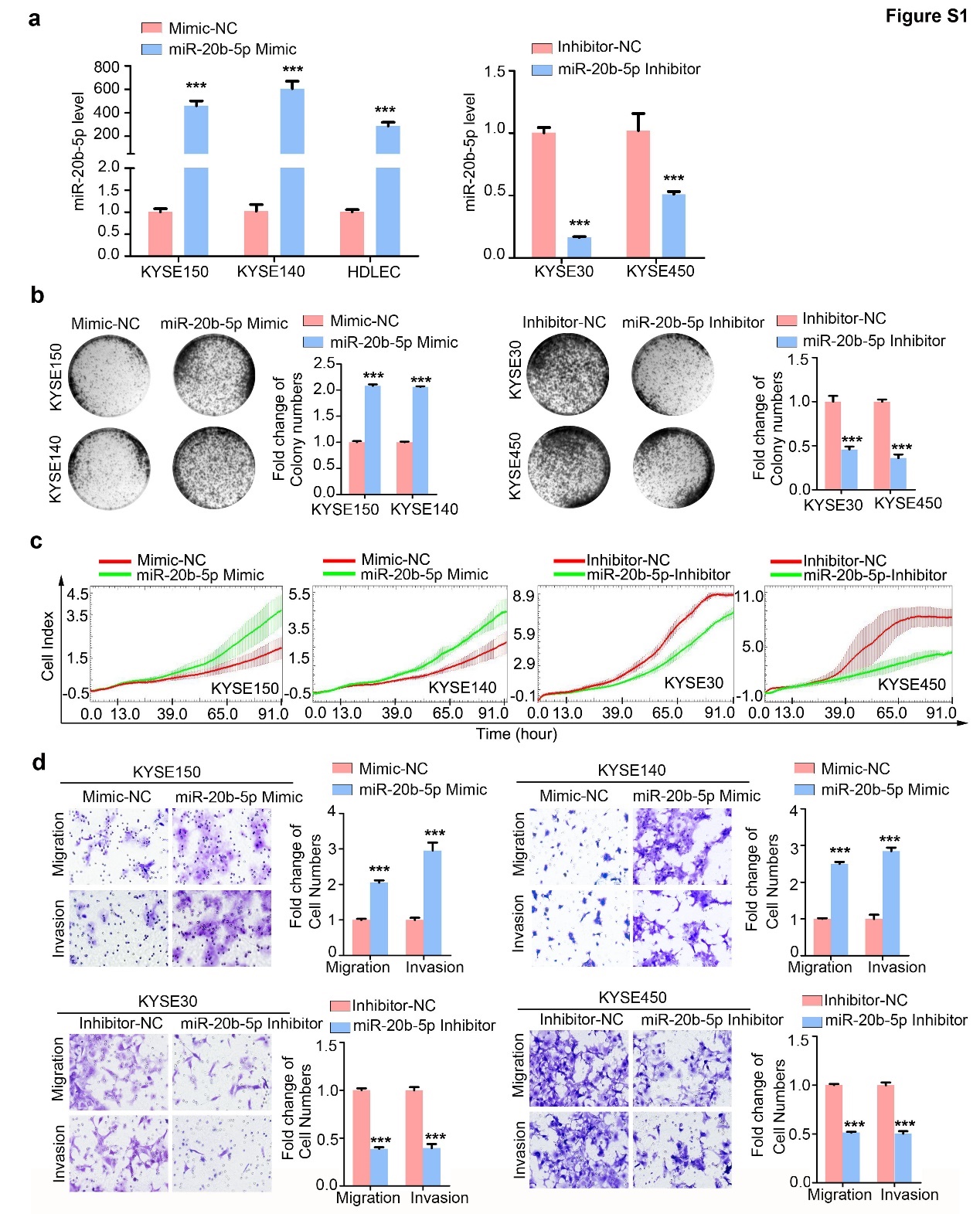


**Figure S1. miR-20b-5p promoted the proliferation, colony formation, invasion and migration abilities of ESCC cells.**

**(a)** qRT-PCR analysis of the miR-20b-5p levels.

KYSE150/KYSE140/HDELC cells were transfected with the miR-20b-5p mimic or the mimic negative control (Mimic-NC) and KYSE30/KYSE450 cells were transfected with the miR-20b-5p inhibitor or inhibitor negative control (Inhibitor-NC).

**(b)** After transfection for 24 h, a colony formation assay was performed to analyze the effects of miR-20b-5p on colony formation ability.

**(c)** After transfection for 24 h, the xCELLigence Real-Time Cell Analyzer (RTCA)-MP system was used to analyze the effects of miR-20b-5p on cell proliferation.

**(d)** After transfection for 48 h, Transwell assays were performed to analyze the effects of miR-20b-5p on cell migration and invasion. The quantitative data are shown as histograms, and the photographs are representative of the migrated/invaded cells. Original magnification, ×100.

The data are representative of three independent experiments. The error bars represent the SEM. ****P* < 0.001; two-tailed unpaired Student’s *t*-test.


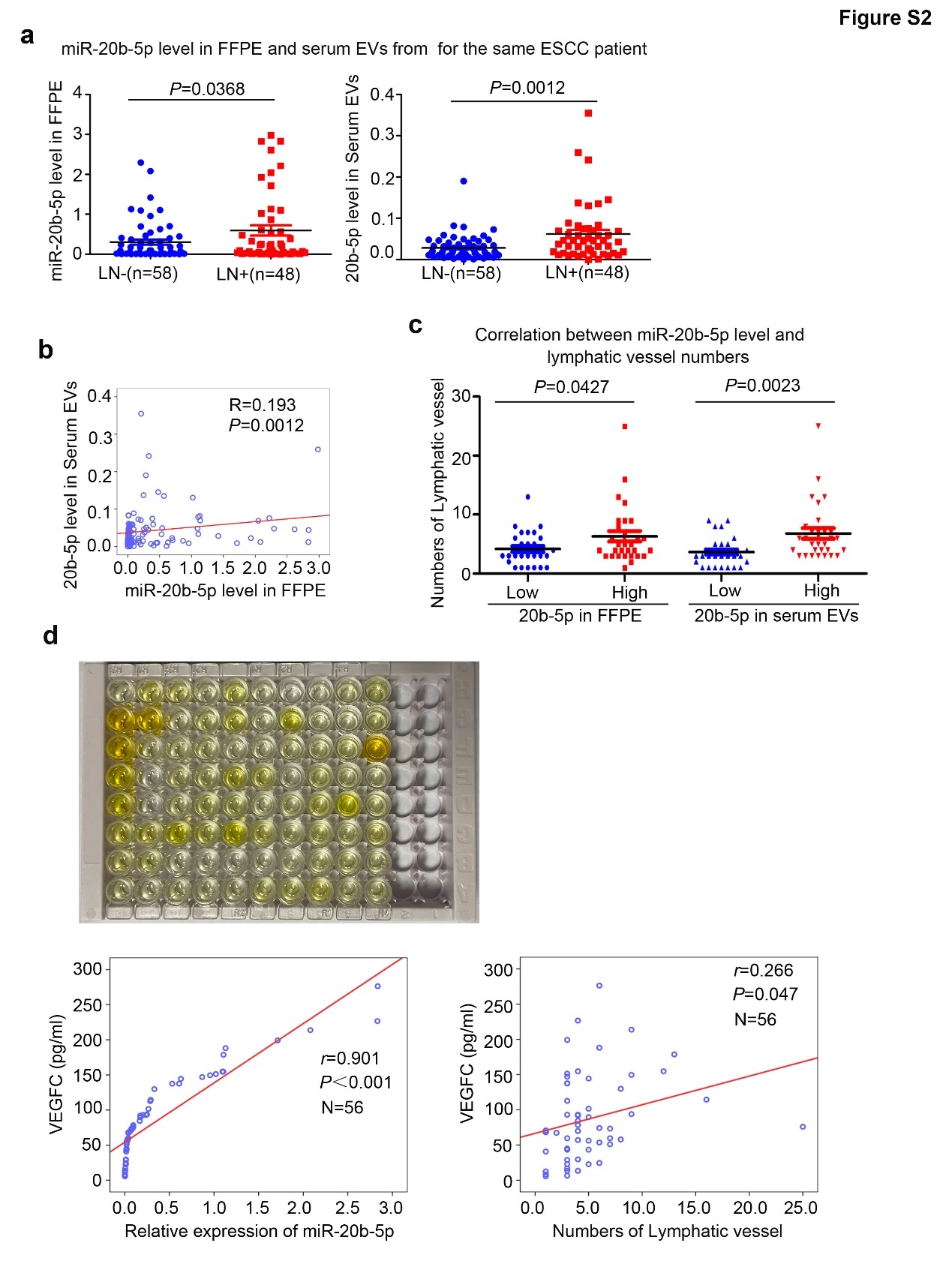


**Figure S2. EV-riched miR-20b-5p is positively correlated with the number and density of lymphatic vessels of ESCC tissues.**

**(a)** qRT-PCR of the miR-20b-5p levels in FFPE samples from ESCC patients with and without LNM. qRT-PCR of the miR-20b-5p levels in serum EVs from ESCC patients with and without LNM. The serum EV miR-20b-5p levels were normalized to the mouse lncRNA GM13008 (exogenous reference).

**(b)** Pearson correlation analysis of the miR-20b-5p levels in serum EVs and FFPE samples from the same patient in (a).

**(c)** Analysis of miR-20b-5p and the numbers of lymphatic vessels in the ESCC FFPE samples from the same patient in (a).

**(d)** Pearson correlation analysis of the VEGFC levels in serum and miR-20b-5p levels, the numbers of lymphatic vessels in ESCC tissues from the same patient in (a).


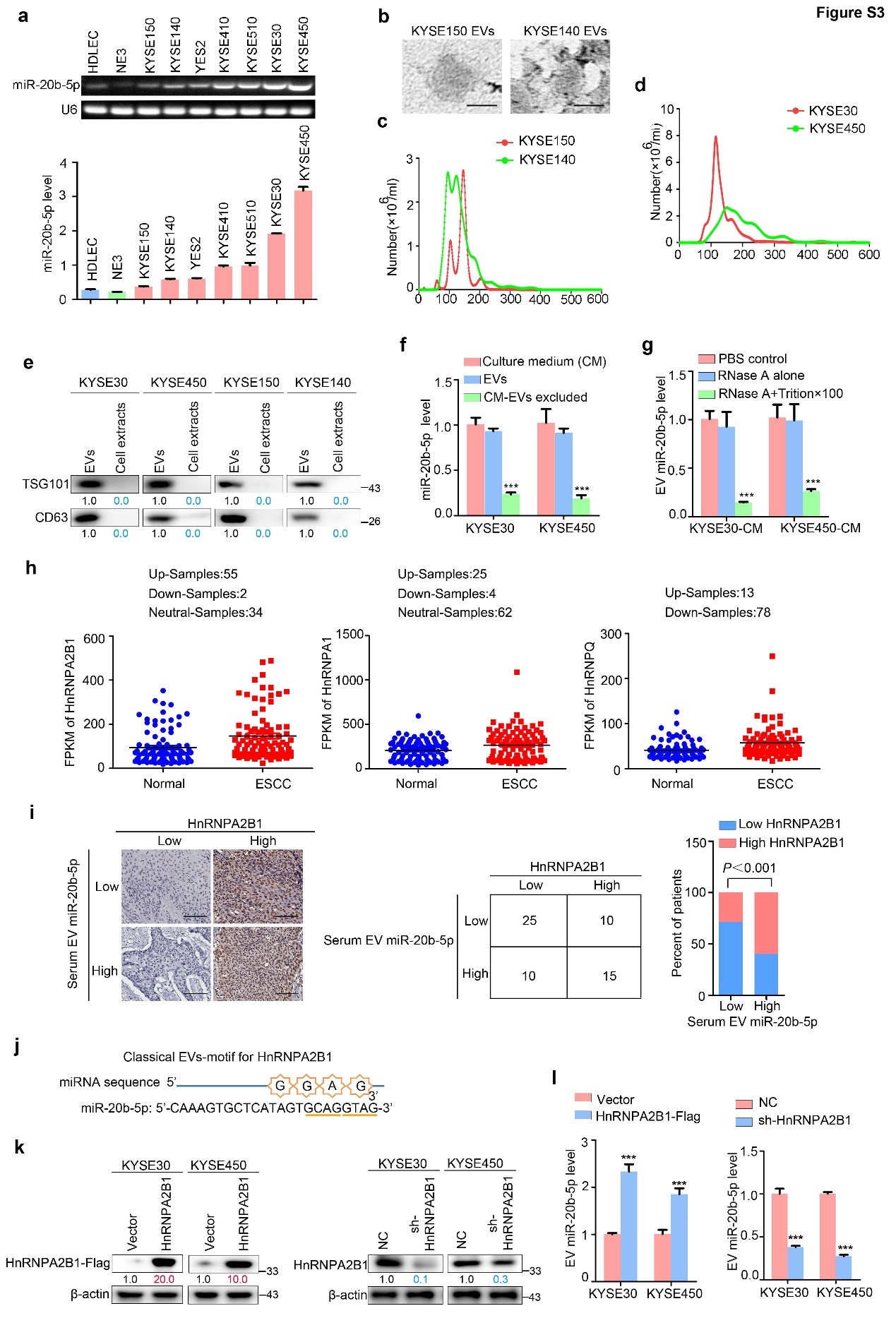


**Figure S3.** **ESCC-derived EVs carry miR-20b-5p into tumor microenvironment.**

**(a)** qRT-PCR analysis of the miR-20b-5p expression levels in seven ESCC cell lines, human immortalized esophageal epithelial cells (NE3) and human dermal lymphatic endothelial cells (HDLECs). Gel electrophoresis of the PCR products amplified by qRT-PCR.

**(b)** Electron microscopy images of EVs secreted by KYSE150 and KYSE140 cells. Scale bar, 100 nm.

**(c-d)** NanoSight particle tracking analysis of the exosome size distributions and numbers.

**(e)** Western blot analysis of the exosomal protein markers TSG101 and CD63 in the EVs and cytoplasmic lysates of KYSE30, KYSE450, KYSE150 and KYSE140 cells.

**(f)** qRT-PCR analysis of the miR-20b-5p levels in EVs, culture medium (CM), and CM devoid of EVs from KYSE30 and KYSE450 cells. The supernatants of KYSE30 and KYSE450 cells were collected and evenly divided them into two parts: one portion was CM, and the other was used for the extraction and concentration of EVs by ultracentrifugation and removal from the CM. RNA was extracted from the CM, from EVs and from the concentrated supernatant devoid of EVs for the detection of miR-20b-5p expression. The expression of miR-20b-5p in EVs was nearly equal to that in CM, while that in the CM without EVs was very low, indicating that the miR-20b-5p in the supernatant was mainly derived from EVs from ESCC cells.

**(g)** qRT-PCR analysis of the miR-20b-5p levels in the CM of KYSE30 and KYSE450 cells treated with RNaseA (2 mg/ml) alone or in combination with Triton X-100 (0.1%) for 30 min. RNase A cannot penetrate the membrane or EVs and can digest only the RNA outside of EVs. After treatment with RNase A, RNA was extracted from the supernatants to detect the expression of miR-20b-5p. The supernatant expression of miR-20b-5p did not significantly differ between the PBS group and the group treated with RNase A alone. Disruption of the EV membrane with Triton X-100 and treatment with RNase A significantly decreased the expression of miR-20b-5p, revealing that the miR-20b-5p in the cell supernatant is mainly derived from EVs.

**(h)** The expression of HnRNPA2B1, HnRNPA1 and HnRNPQ in 91 ESCC tumors and adjacent normal tissues was analyzed by transcript sequencing.

**(i)** Representative IHC staining of HnRNPA2B1 in ESCC FFPE tissues. Scale bar, 100μm.Percentages of patients with high HnRNPA2B1 levels and low HnRNPA2B1 expression levels according to serum EV miR-20b-5p levels from the same patient in Figure S2a.

**(j)** Two similar sequences at the 3' end of miR-20b-5p to GGAG (hEV motif) mediates its binding to the RNA-binding protein hnRNPA2B1 and controls the sorting of such miRNAs into EVs.

**(k)** Western blot analysis of the hnRNPA2B1 in the KYSE30 and KYSE450 cells after transfection with a control or hnRNPA2B1 plasmid (HnRNPA2B1 gene were fused with Flag tag). The overexpression efficiency was detected by WB using Flag antibody. Western blot analysis of the hnRNPA2B1 in the KYSE30 and KYSE450 cells after transfection with control or hnRNPA2B1 shRNA. The knockdown efficiency was detected by WB using HnRNPA2B1 antibody.

**(l)** qRT-PCR analysis of the miR-20b-5p levels in EVs derived from KYSE30 and KYSE450 cells after transfection with a control or hnRNPA2B1 plasmid/shRNA.

**
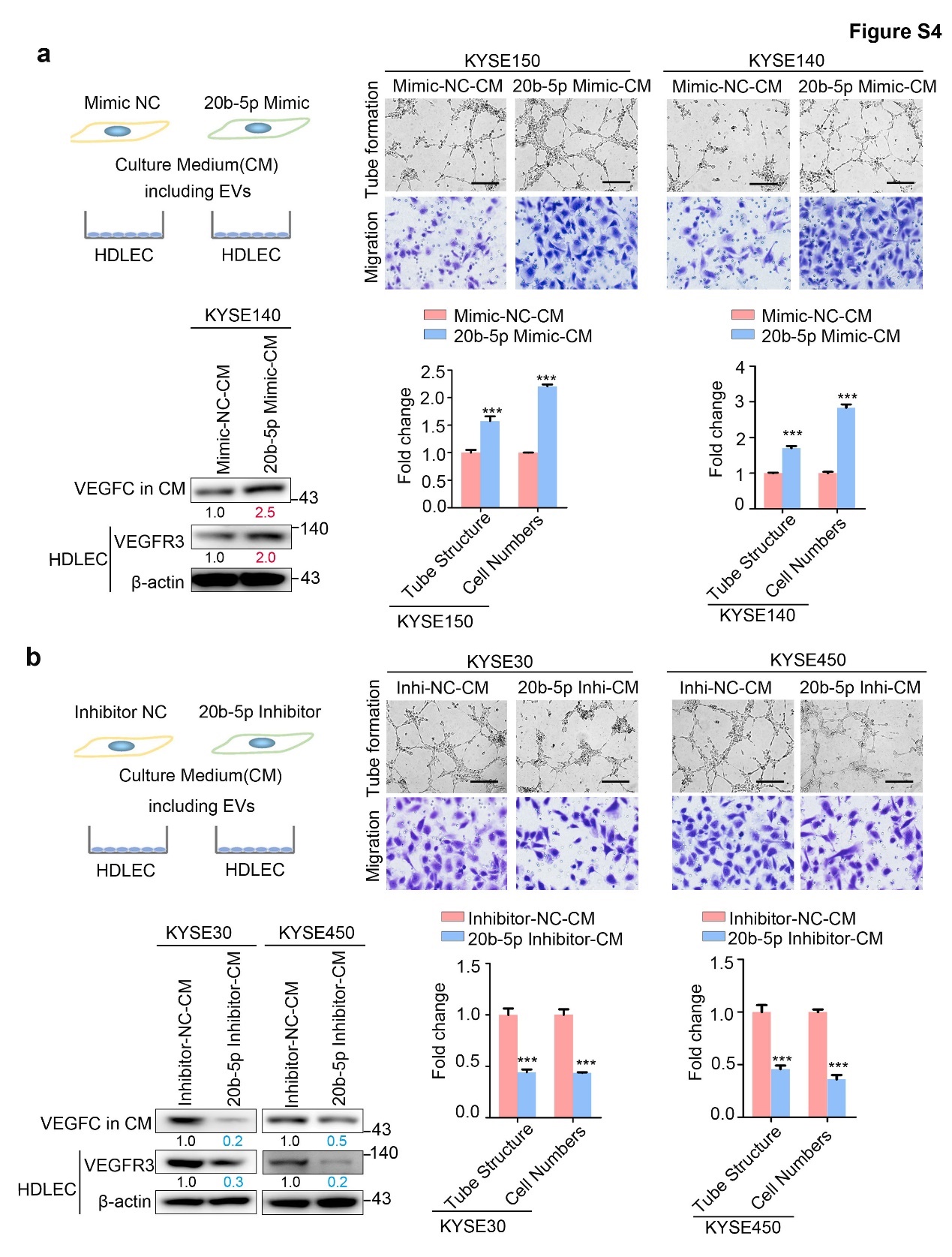
**

**Figure S4.** **miR-20b-5p promotes the migration and tube formation of HDLECs *in vitro.***

1. Representative images of HDLECs cultured with CM (CM including EVs) from KYSE150/KYSE140 miR-20b-5p-overexpressing cells and corresponding control cells. Matrigel tube formation assay (scale bar, 2mm) and migration assay (Original magnification, ×100). Western blot analysis of VEGFC in CM and VEGFR3 in HDLECs.
2. Representative images of HDLECs cultured with CM (CM including EVs) from KYSE30/KYSE450miR-20b-5p-silenced cells and corresponding control cells. Matrigel tube formation assay (Scale bar, 2mm) and migration assay. Western blot analysis of VEGFC in CM and VEGFR3 in HDLECs.

**
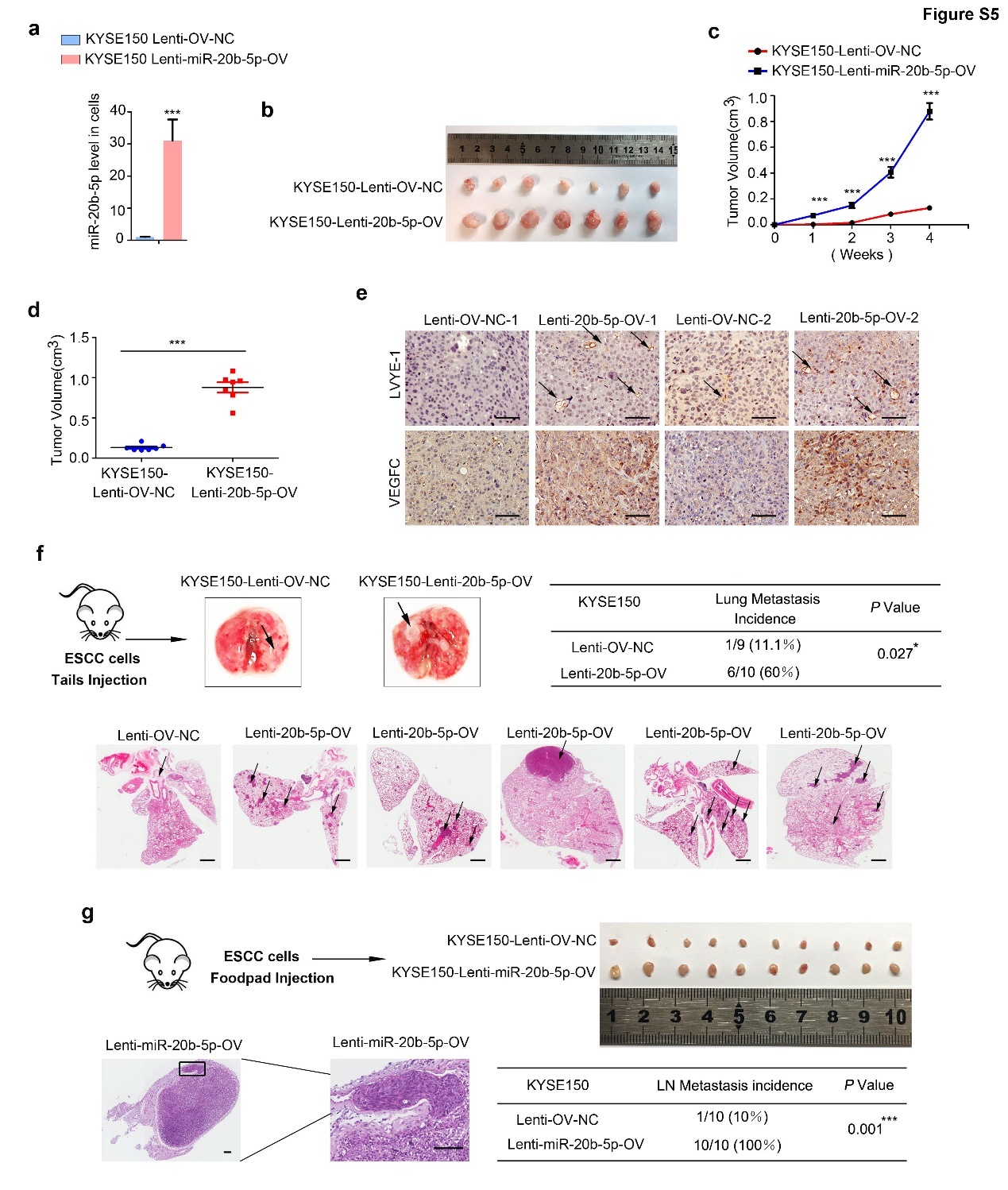
**

**Figure S5. MiR-20b-5p promotes ESCC growth, lung metastasis and lymphatic metastasis *in vivo*.**

**(a)** KYSE150 cells were stably infected with lentiviral vectors expressing control (KYSE150-Lenti-OV-NC) or miR-20b-5p (KYSE150-Lenti-miR-20b-5p-OV), and the miR-20b-5p levels were analyzed by qPCR.

**(b-e)** The top backs of the mice were injected subcutaneously with 5×10^5^ KYSE150-Lenti-miR-OV-NC or KYSE150-Lenti-miR-20b-5p-OV cells in 0.1 mL. The growth of both control and Lenti-miR-20b-5p cell-induced tumors in nude mice was measured weekly (n=7).

**(b)** The images of the ultimately formed tumors.

**(c)** Tumor growth curve analysis of mice bearing Lenti-miR-20b-5p-OV cells compared to mice bearing control cells.

**(d)** Tumor mass analysis.

**(e)** Representative IHC staining of LYVE-1 and VEGFC in KYSE150 xenografts with the indicated treatments. Scale bar, 100μm.

**(f)** Rate of lung metastasis after the tail vein injection of KYSE150-Lenti-OV-NC or KYSE150-Lenti-miR-20b-5p-OV cells. Lung metastasis of representative tumors was determined by HE analysis. Scale bar, 1mm.

**(g)** Volumes of popliteal lymph nodes and rates of lymphatic metastasis after foot pad injections of either KYSE150-Lenti-OV-NC or KYSE150-Lenti-miR-20b-5p-OV cells. Lymphatic metastasis of representative tumors was determined by HE analysis. Scale bar, 100μm.

The statistical analyses were performed using the two-tailed Student’s t-test. **P* < 0.05, ****P* < 0.001.

**
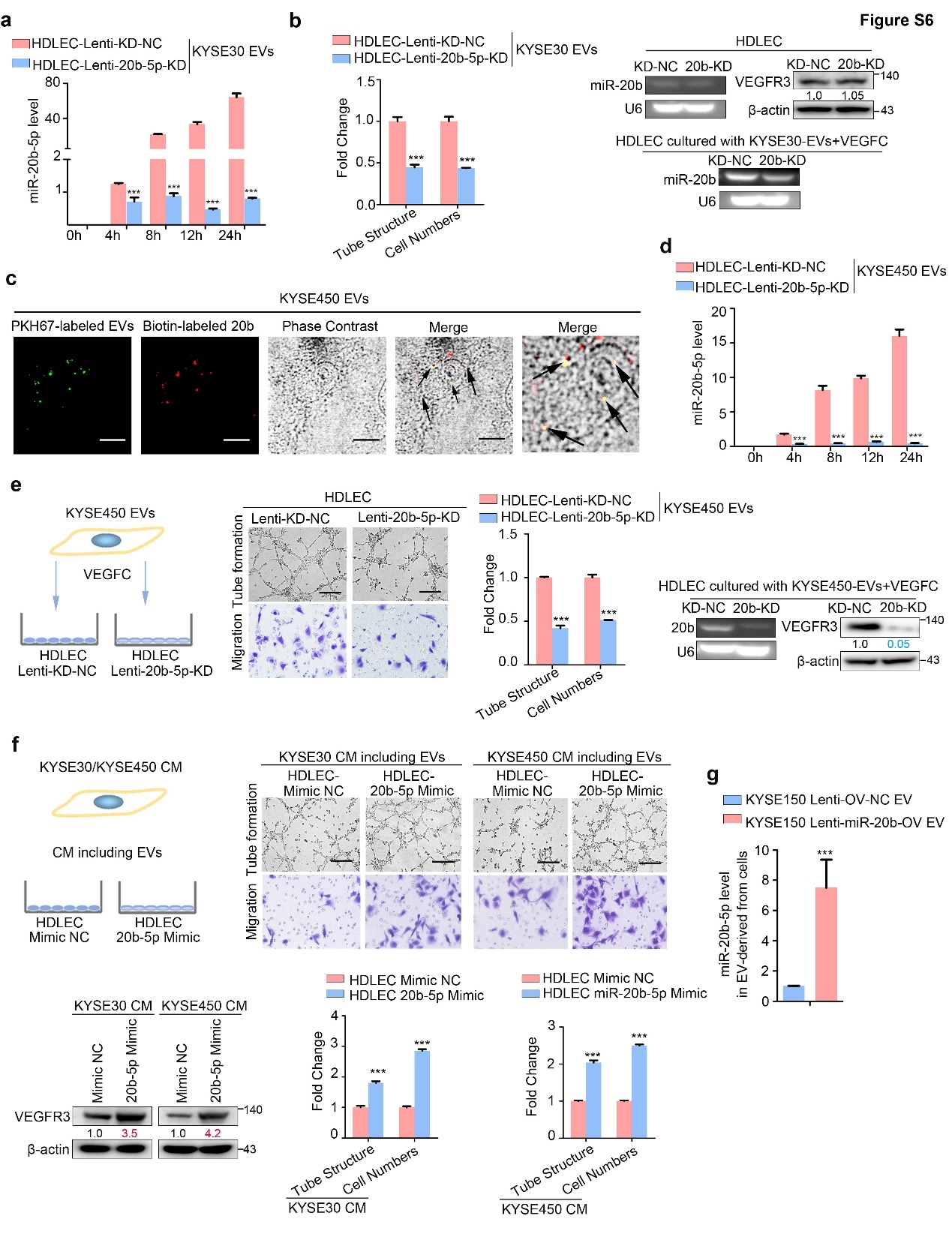
**

**Figure S6.** **EV-riched miR-20b-5p induces lymphangiogenesis of HDLECs *in vitro*.**

**(a)** HDLECs were incubated with PKH67-labeled EVs carried biotin-miR-20b-5p from KYSE30. qRT-PCR analysis of the miR-20b-5p levels in HDLEC.

**(b)** HDLEC-Lenti-miR-20b-5p-KD or HDLEC-Lenti-KD-NC were cultured with EVs from KYSE30 cells. Gel electrophoresis of the miR-20b-5p PCR products amplified by qRT-PCR. Western blot analysis of VEGFR3 in HDLECs.

**(c)** Fluorescence observation of HDLEC cells at 24 h after incubation with PKH67-labeled (green) EVs carried biotin-miR-20b-5p (red) from KYSE450. Representative fluorescence images and phase-contrast images are shown. Scale bar, 30μm.

**(d)** qRT-PCR analysis of the miR-20b-5p levels in (c).

**(e)** Representative images of HDLEC-Lenti-miR-20b-5p-KD or HDLEC-Lenti-KD-NC cultured with EVs from KYSE450 cells. Matrigel tube formation assay (Scale bar, 2mm) and migration assay (Original magnification, ×100). Gel electrophoresis of the miR-20b-5p PCR products amplified by qRT-PCR. Western blot analysis of VEGFR3 in HDLECs.

**(f)** Representative images of HDLECs transfected with the miR-20b-5p mimic or control and cultured with CM from KYSE30/KYSE450 cells. Matrigel tube formation assay (Scale bar, 2mm) and migration assay (Original magnification, ×100). Western blot analysis of VEGFR3 in HDLECs.

**(g)** miR-20b-5p levels in EVs from KYSE150-Lenti-OV-NC and KYSE150-Lenti-miR-20b-5p-OV cells were analyzed by qPCR.

The data are representative of three independent experiments. The error bars represent the SEM. ****P* < 0.001; two-tailed unpaired Student’s t-test.

**
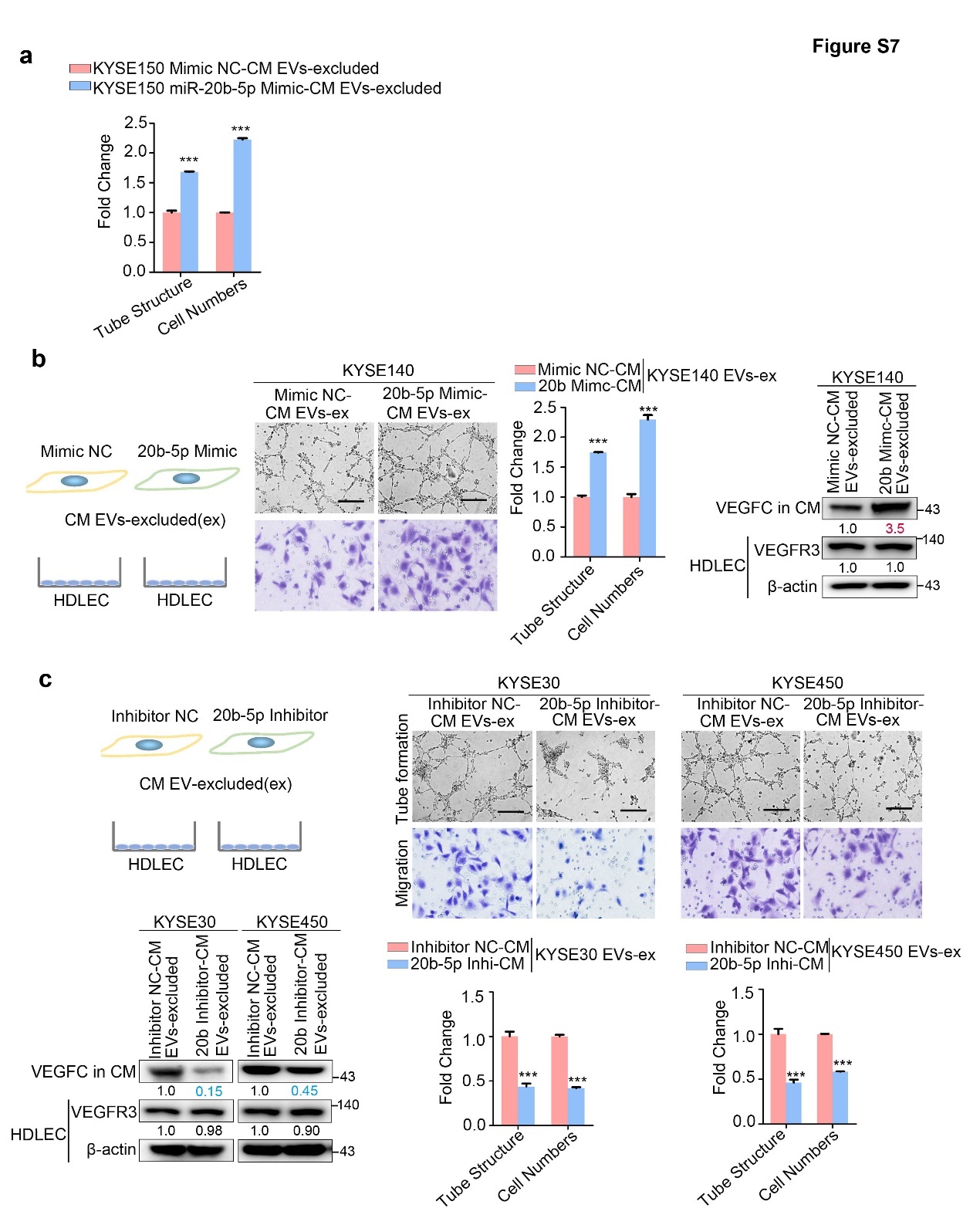
**

**Figure S7. miR-20b-5p induces lymphangiogenesis of HDLECs by affecting the ESCC cell secretion of VEGFC.**

**(a)** HDLECs cultured with CM (devoid of EVs) from KYSE150 miR-20b-5p-overexpressing cells and corresponding control cells. The quantitative data are shown as histograms.

**(b)** Representative images of HDLECs cultured with CM (devoid of EVs) from KYSE140 miR-20b-5p-overexpressing cells and corresponding control cells. Matrigel tube formation assay (Scale bar, 2mm) and migration assay (Original magnification, ×100). Western blot analysis of VEGFC in CM and VEGFR3 in HDLECs.

**(c)** Representative images of HDLECs cultured with CM (devoid of EVs) from KYSE30/KYSE450 miR-20b-5p-silenced cells and corresponding control cells. Matrigel tube formation assay (Scale bar, 2mm) and migration assay (Original magnification, ×100). Western blot analysis of VEGFC in CM and VEGFR3 in HDLECs.

The data are representative of three independent experiments. The error bars represent the SEM. ****P* < 0.001; two-tailed unpaired Student’s t-test.


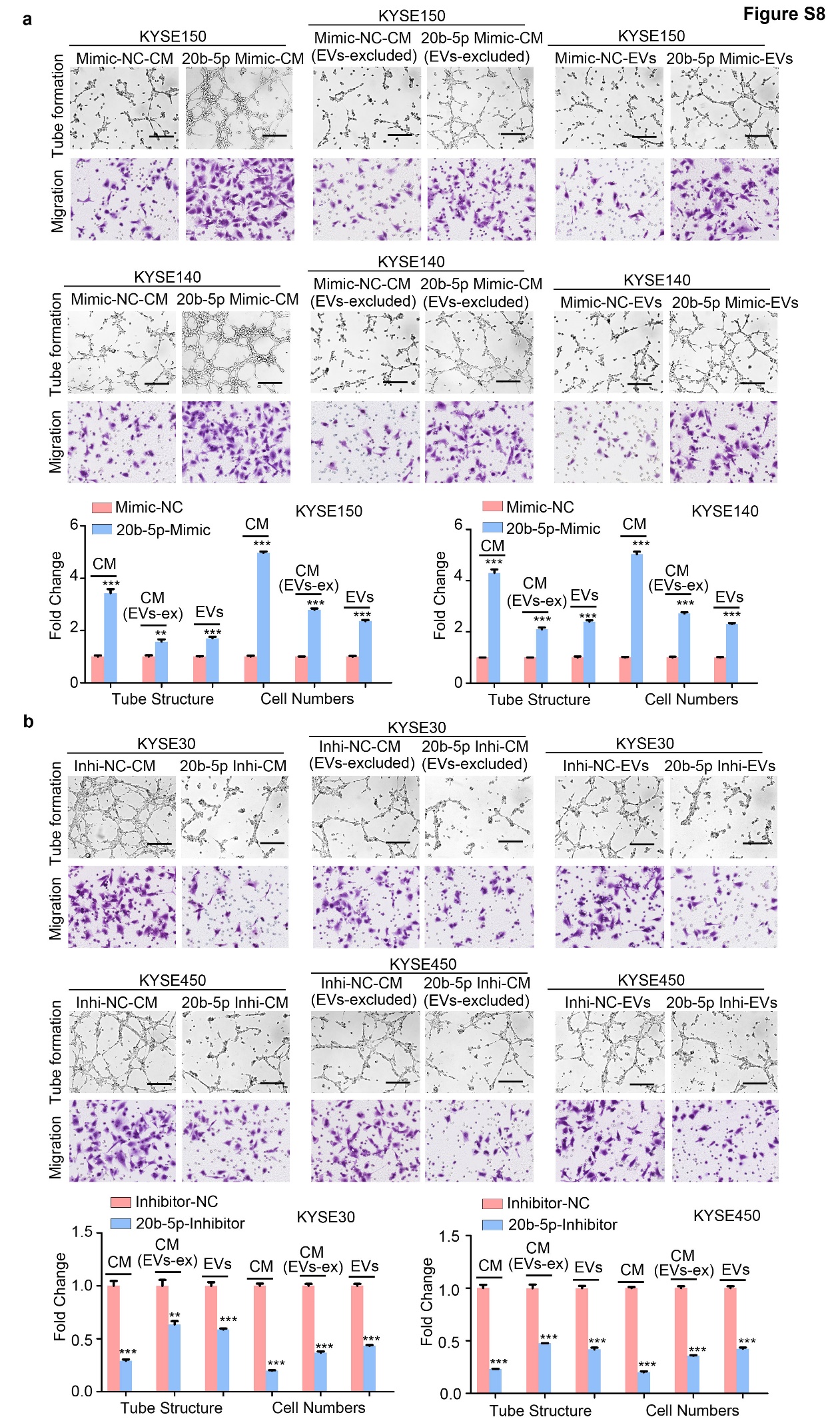


**Figure S8. miR-20b-5p in ESCC cells and EVs-riched miR-20b-5p derived from ESCC cells have an additive effect on the migration and tube formation abilities of the HDLECs.**

The supernatants of KYSE30/KYSE450 cells were evenly divided them into two parts: one portion was CM, and the other was used for the extraction and concentration of EVs by ultracentrifugation and removal from the medium.

**(a)** Representative images of HDLECs cultured with conditioned medium (CM, CM removing EVs, EVs) from KYSE150/KYSE140 miR-20b-5p-overexpressing cells and corresponding control cells. Matrigel tube formation assay (Scale bar, 2mm) and migration assay (Original magnification, ×100).

**(b)** Representative images of HDLECs cultured with conditioned medium (CM, CM removing EVs, EVs) from KYSE30/KYSE450 miR-20b-5p-silenced cells and corresponding control cells. Matrigel tube formation assay (Scale bar, 2mm) and migration assay (Original magnification, ×100).

The data are representative of three independent experiments. The error bars represent the SEM. ***P* < 0.01, ****P* < 0.001; two-tailed unpaired Student’s t-test.


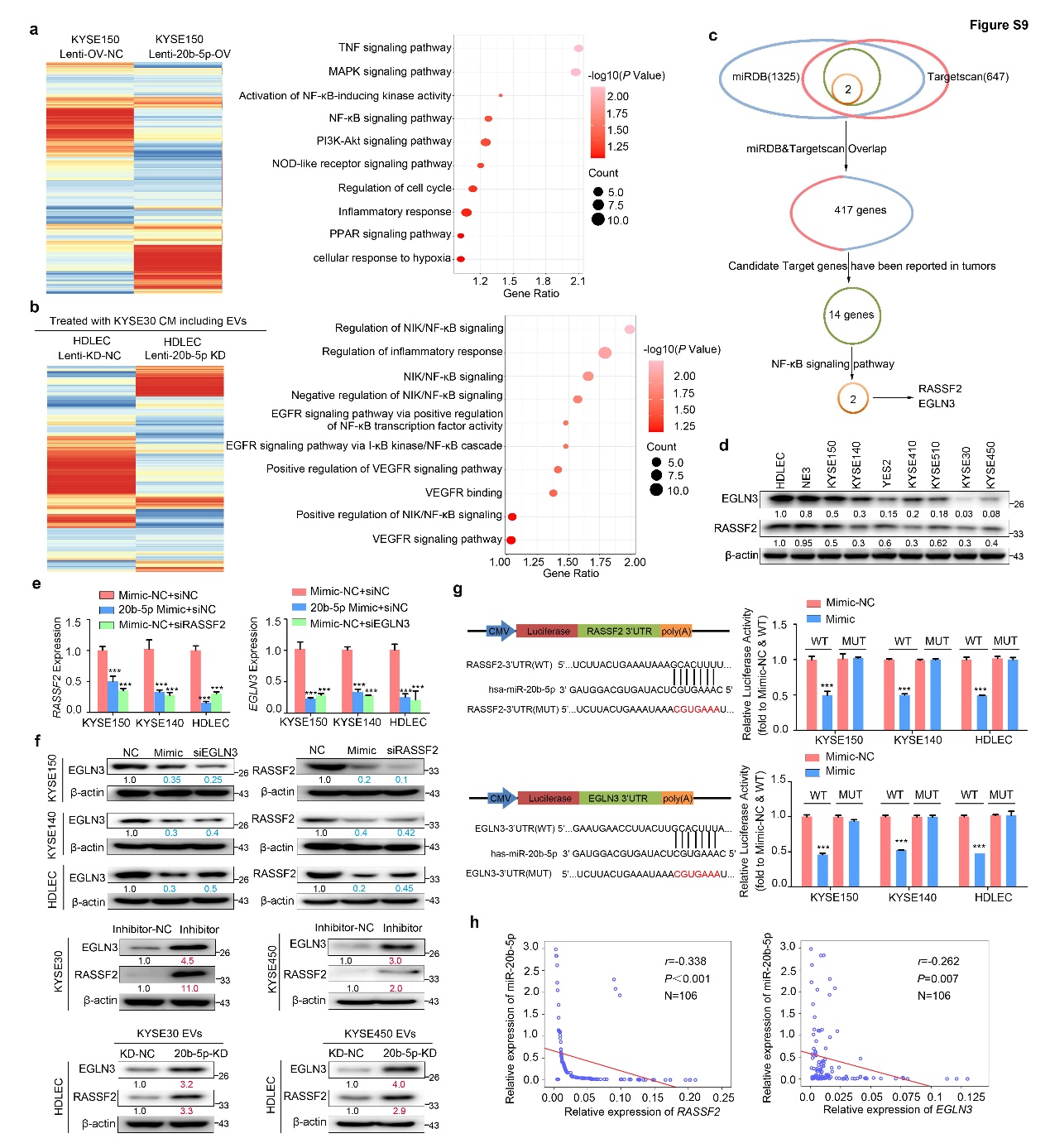


**F****igure S9. RASSF2 and EGLN3 are two direct targets of miR-20b-5p.**

1. Left: Cluster analysis of deregulated genes in KYSE150-Lenti-OV-NC and KYSE150-Lenti-miR-20b-5p-OV. Right: Differential gene enrichment analysis.
2. Left: Cluster analysis of deregulated genes in HDLEC-Lenti-miR-20b-5p-KD and HDLEC-Lenti-KD-NC and cultured with CM (including EVs) from KYSE30. Right: Differential gene enrichment analysis. The sample size is 1 replication for each cell line in (a) and (b).
3. Venn diagram of putative miR-20b-5p target genes.
4. Western blot analysis of the RASSF2 and EGLN3.
5. qPCR analysis of RASSF2 and EGLN3 in ESCC cells treated with the miR-20b-5p mimic, RASSF2/EGLN3 siRNA, or the corresponding control.
6. Western blot analysis of RASSF2 and EGLN3 in ESCC cells treated with the miR-20b-5p mimic, RASSF2/EGLN3 siRNA, miR-20b-5p inhibitor or the corresponding control. Western blot analysis of RASSF2 and EGLN3 in HDLEC-Lenti-miR-20b-5p-KD and HDLEC-Lenti-KD-NC cultured with EVs.
7. The diagram shows the regions containing the miR-20b-5p-binding site on the luciferase reporter constructs containing the 3’ UTRs of RASSF2 and EGLN3. Cells were transfected with the WT or MUT luciferase vector and the miR-20b-5p mimic or mimic-NC. Luciferase reporter activity was normalized to that of Renilla.
8. Pearson correlation analysis of miR-20b-5p and the RASSF2/EGLN3 mRNA levels in ESCC FFPE samples from the same patient in Figure S2a.

The data are representative of three independent experiments. The statistical analyses were performed using the two-tailed unpaired Student’s t-test (e) and Pearson’s Χ2-test (h). ****P* < 0.001.


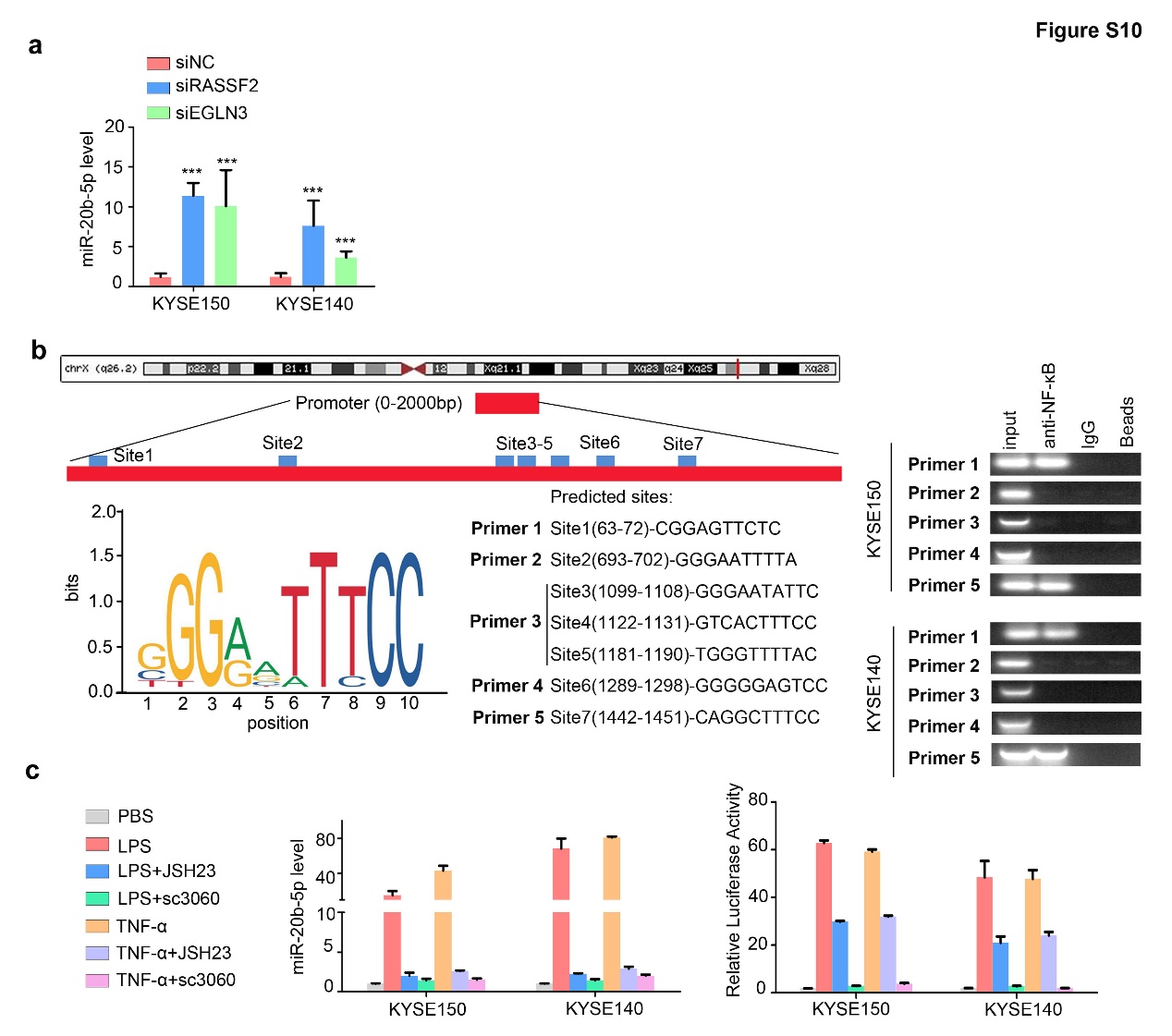


**Figure S10. NF-κB transcriptionally regulates the miR-20b-5p promoter status.**

1. qPCR analysis of miR-20b-5p in cells transfected with RASSF2/EGLN3 siRNA and the corresponding controls.
2. Analysis of possible physical associations between miR-20b-5p promoter regions and NF-κB p65. ChIP assay of the effect of NF-κB p65 on miR-20b-5p relative to IgG. Gel electrophoresis was performed on the PCR products from the ChIP assay.
3. qRT-PCR analysis of miR-20b-5p levels and luciferase reporter assay of cells transfected with the NF-κB response element plasmid in KYSE150/KYSE140 treated with LPS (1μg/ml)/TNF-α (10ng/ml) alone or in combination with JSH23 (5μM)/sc-3060 (10μM).

The data are representative of three independent experiments. The error bars represent the SEM. ****P* < 0.001; two-tailed unpaired Student’s t-test.


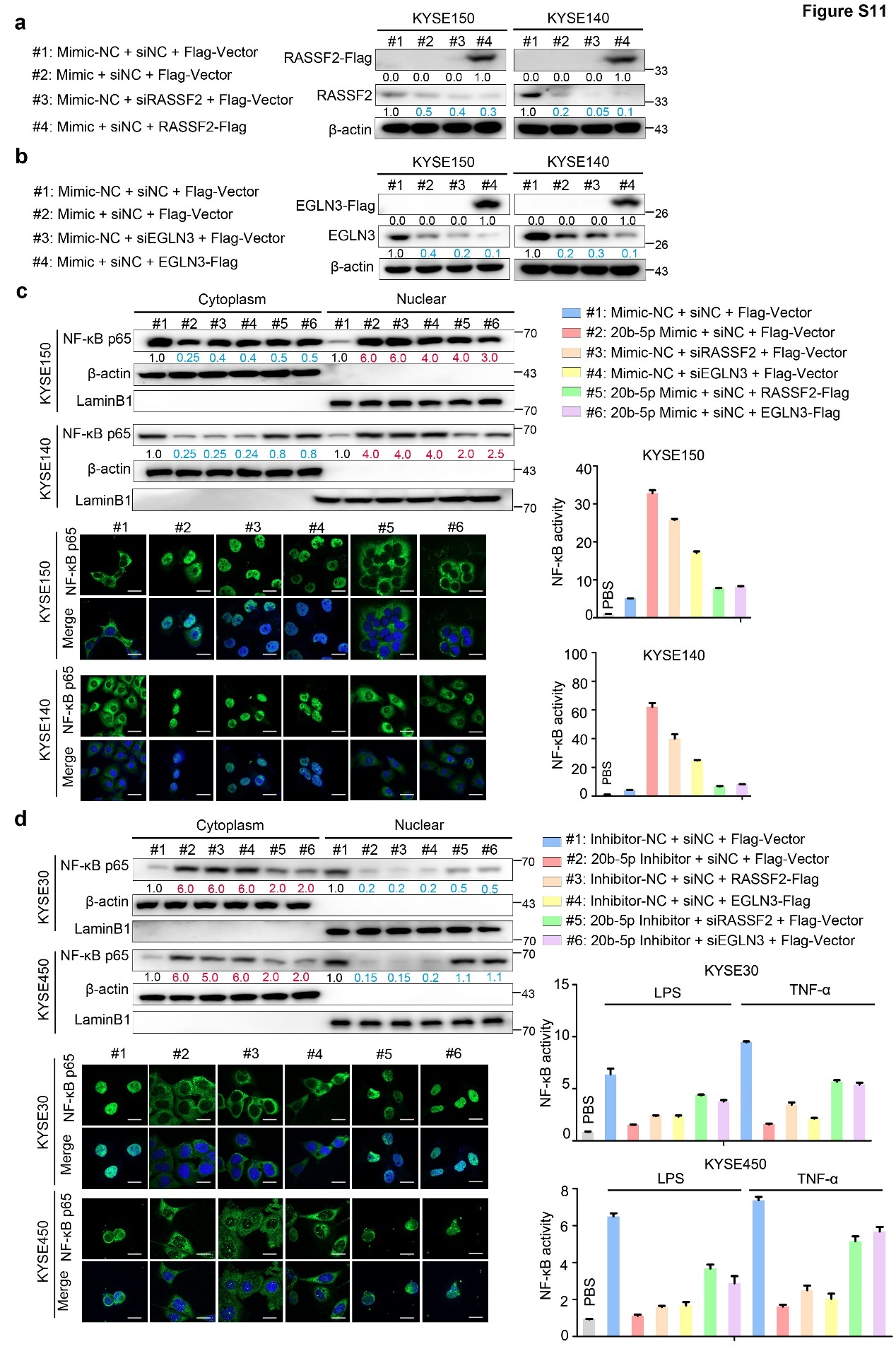


**Figure S11. MiR-20b-5p promotes the nuclear entry of NF-κB p65 in ESCC.**

**(a-b)** Western blot analysis of the indicated proteins in cells transfected with the miR-20b-5p mimic, RASSF2/EGLN3 siRNA, or RASSF2/EGLN3 plasmid and the corresponding controls.

**(c-d)** Western blot analysis of p65 in nuclear and cytoplasmic lysate fractions. Immunofluorescence analysis of NF-κB sublocalization (green) as determined by confocal microscopy (Scale bar, 30μm). Luciferase reporter assay of cells transfected with the pNF-κB-luc (NF-κB response element) plasmid.

**(c)** KYSE150/KYSE140 cells were transfected with the miR-20b-5p mimic or RASSF2/EGLN3 siRNA or cotransfected with the miR-20b-5p mimic and the RASSF2/EGLN3 plasmid.

**(d)** KYSE30/KYSE450 cells were transfected with the miR-20b-5p inhibitor or RASSF2/EGLN3 plasmid or cotransfected with the miR-20b-5p inhibitor and RASSF2/EGLN3 siRNA.

Luciferase reporter activity was normalized to that of Renilla (c-d). The data are representative of three independent experiments. The error bars represent the SEM. ***P* < 0.01, ****P* < 0.001; two-tailed unpaired Student’s t-test.


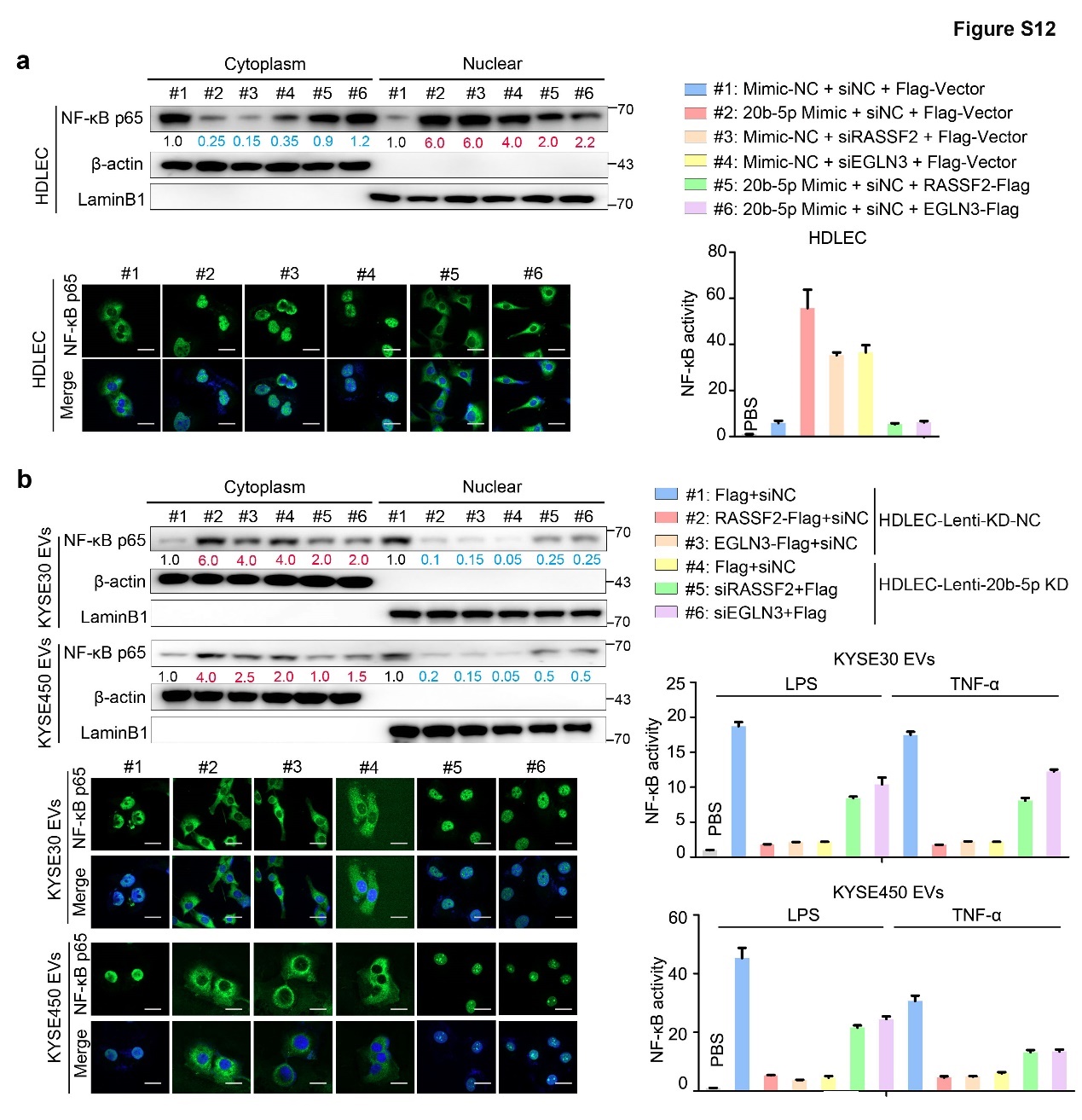


**Figure S12. EV miR-20b-5p promotes the nuclear entry of NF-κB p65 in HDLEC.**

**(a-b)** Western blot analysis of p65 in nuclear and cytoplasmic lysate fractions. Immunofluorescence analysis of NF-κB sublocalization (green) as determined by confocal microscopy (Scale bar, 30μm). Luciferase reporter assay of cells transfected with the NF-κB response element plasmid.

**(a)** HDLECs were transfected with the miR-20b-5p mimic or RASSF2/EGLN3 siRNA or cotransfected with the miR-20b-5p mimic and the RASSF2/EGLN3 plasmid.

**(b)** HDLEC-Lenti-miR-20b-5p-KD were transfected with RASSF2/EGLN3 siRNA and HDLEC-Lenti-KD-NC cells were transfected with the RASSF2/EGLN3 plasmid. The indicated cells were treated with EVs from KYSE30/KYSE450.

Luciferase reporter activity was normalized to that of Renilla (a-b). The data are representative of three independent experiments. The error bars represent the SEM. ***P* < 0.01, ****P* < 0.001; two-tailed unpaired Student’s t-test.

**
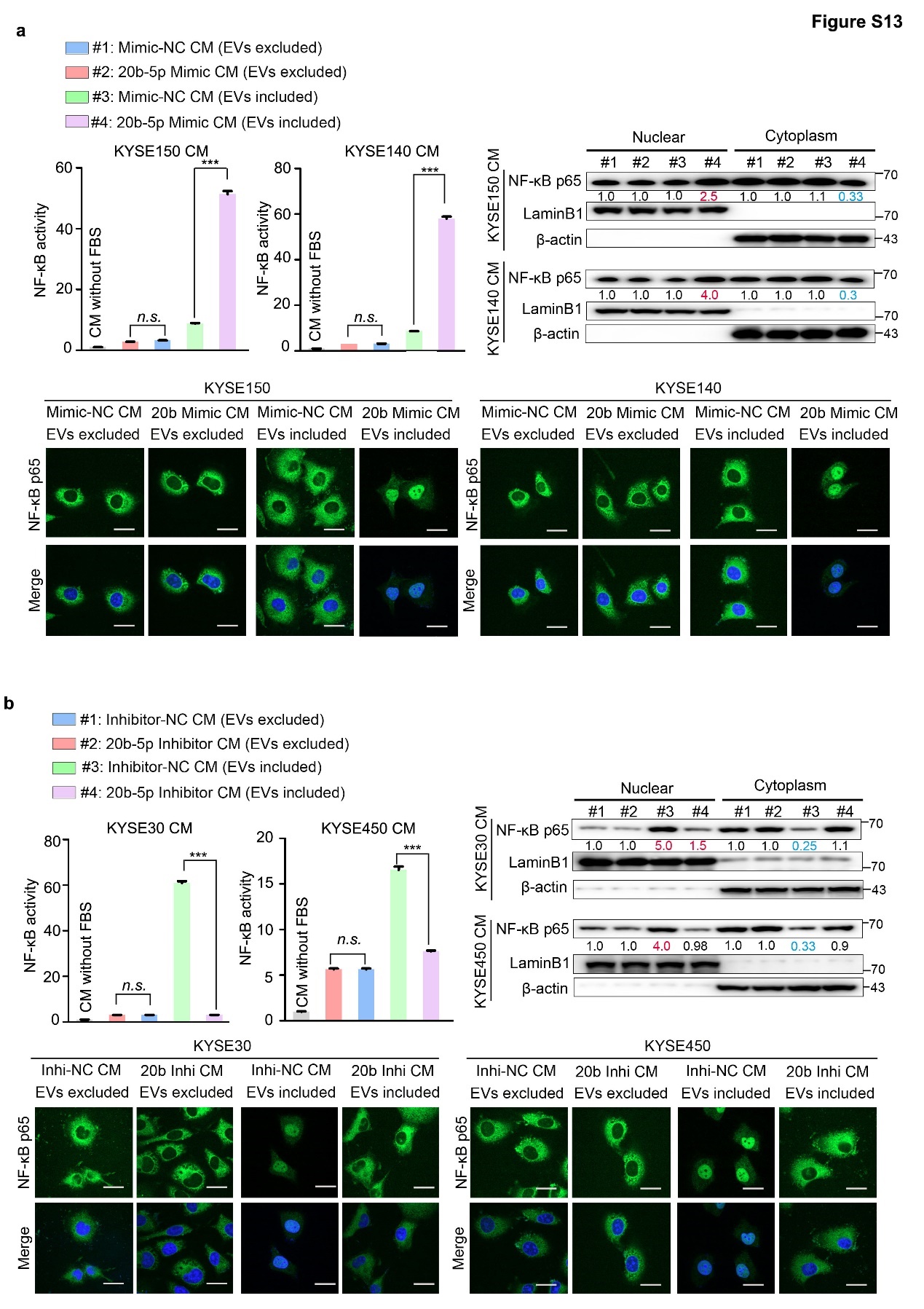
**

**Figure S13. The activation status of NF-κB signaling in HDLECs treated with CM devoid of EVs.**

**(a-b)** Luciferase reporter assay of cells transfected with the NF-κB response element plasmid. Western blot analysis of p65 in nuclear and cytoplasmic lysate fractions. Immunofluorescence analysis of NF-κB sublocalization (green) as determined by confocal microscopy (Scale bar, 30μm).

**(a)** HDLECs cultured with conditioned medium with and without EVs from KYSE150/KYSE140 miR-20b-5p-overexpressing cells and corresponding control cells. **(b)** HDLECs cultured with conditioned medium with and without EVs from KYSE30/KYSE450 miR-20b-5p-silenced cells and corresponding control cells.

Luciferase reporter activity was normalized to that of Renilla. The data are representative of three independent experiments. The error bars represent the SEM. ****P* < 0.001; two-tailed unpaired Student’s t-test.

**
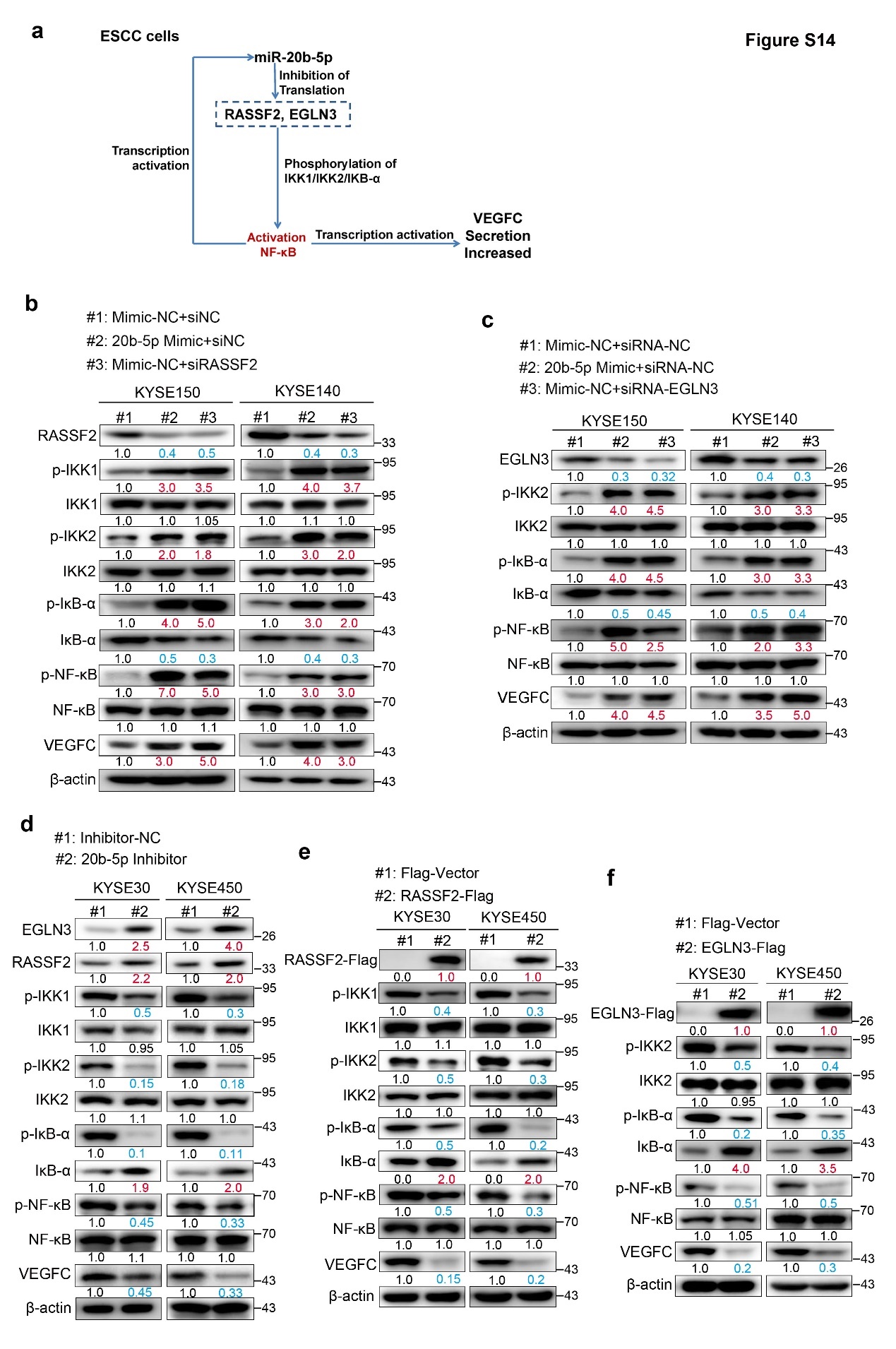
**

**Figure S14.** **MiR-20b-5p transactivates VEGFC by targeting RASSF2/EGLN3-NF-κB signaling pathway.**

1. Schematic model presenting the mechanism of miR-20b-5p in ESCC cells.
2. Western blot analysis of the indicated proteins in KYSE150/KYSE140 cells transfected with the miR-20b-5p mimic, RASSF2 siRNA, and the corresponding control.
3. Western blot analysis of the indicated proteins in KYSE150/KYSE140 cells transfected with the miR-20b-5p mimic, EGLN3 siRNA, and the corresponding control.
4. Western blot analysis of the indicated proteins in KYSE30/KYSE450 cells transfected with the miR-20b-5p inhibitor and the corresponding control.
5. Western blot analysis of the indicated proteins in KYSE30/KYSE450 cells transfected with the RASSF2 plasmid and the corresponding control.
6. Western blot analysis of the indicated proteins in KYSE30/KYSE450 cells transfected with the EGLN3 plasmid and the corresponding control.


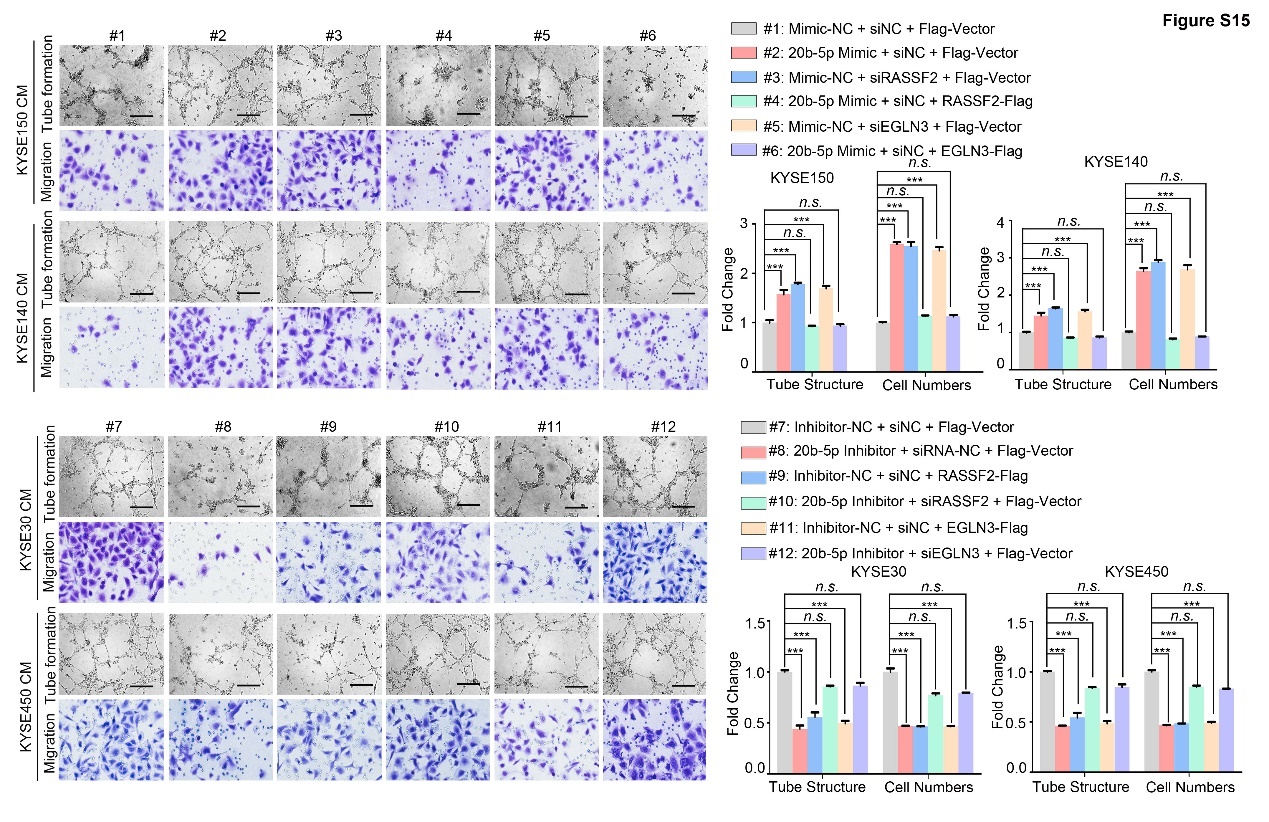


**Figure S15. RASSF2 and EGLN3 mediate the effects of miR-20b-5p on HDLEC** **migration and tube formation.**

KYSE150/KYSE140 cells were transfected with the miR-20b-5p mimic or RASSF2/EGLN3 siRNA or cotransfected with the miR-20b-5p mimic and the RASSF2/EGLN3 plasmid. KYSE30/KYSE450 cells were transfected with the miR-20b-5p inhibitor or the RASSF2/EGLN3 plasmid or cotransfected with the miR-20b-5p inhibitor and RASSF2/EGLN3 siRNA. Representative images of HDLECs cultured with CM (including EVs) from the indicated cells and control cells. Matrigel tube formation assay (Scale bar, 2mm) and migration assay (Original magnification, ×100).

The data are representative of three independent experiments. The error bars represent the SEM. ****P* < 0.001, two-tailed unpaired Student’s *t*-test.


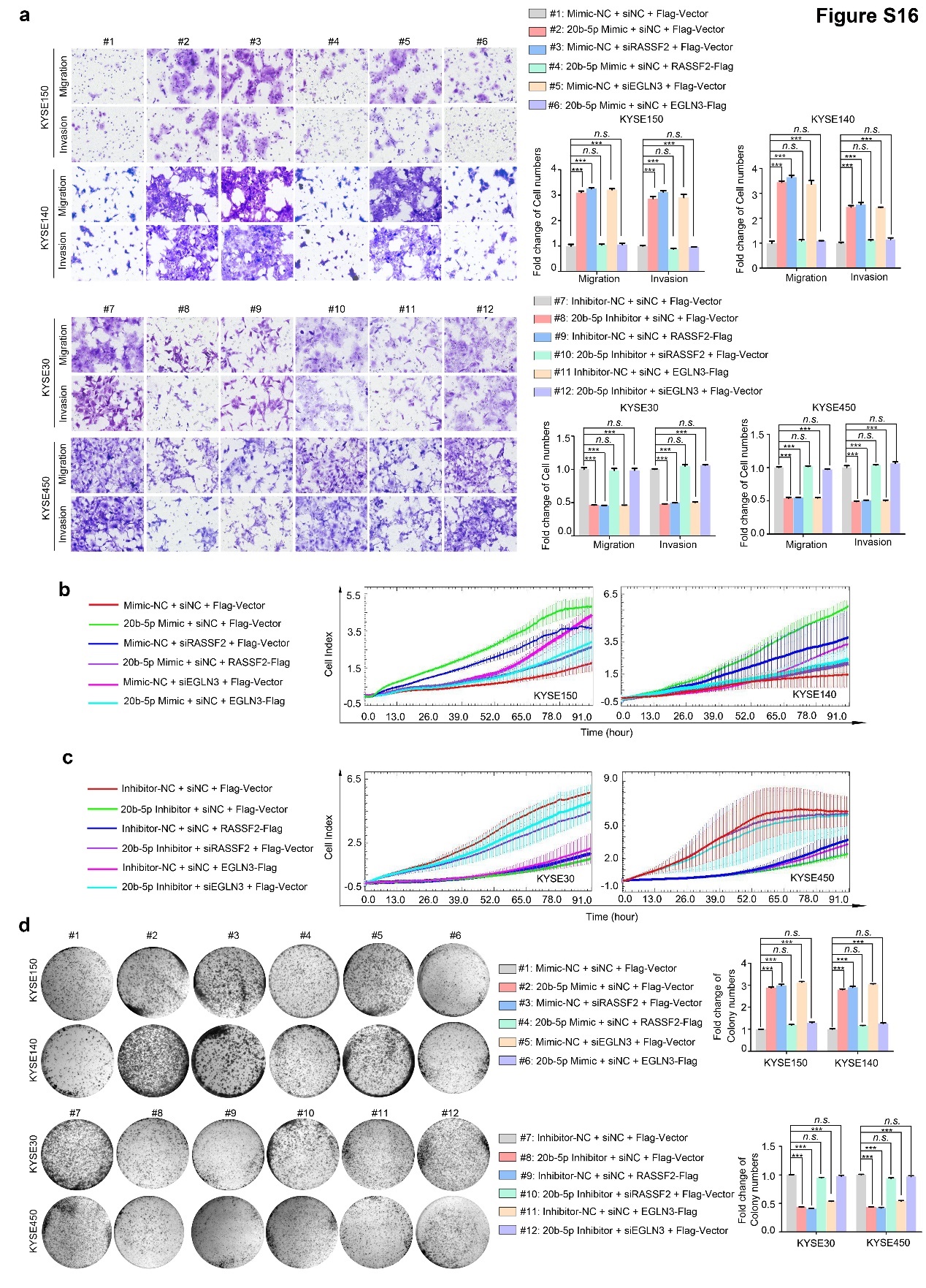


**Figure S16.** **RASSF2 and EGLN3 mediate the effects of miR-20b-5p on ESCC.**

**(a-d)** KYSE150/KYSE140 cells were transfected with the miR-20b-5p mimic or RASSF2/EGLN3 siRNA or cotransfected with the miR-20b-5p mimic and the RASSF2/EGLN3 plasmid. KYSE30/KYSE450 cells were transfected with the miR-20b-5p inhibitor or the RASSF2/EGLN3 plasmid or cotransfected with the miR-20b-5p inhibitor and RASSF2/EGLN3 siRNA.

**(a)** Transwell assays. Original magnification, ×100.

**(b-c)** Growth ability analysis with the xCELLigence Real-Time Cell Analyzer (RTCA)-MP system.

**(d)** Colony formation analysis.

The data are representative of three independent experiments. The error bars represent the SEM. ****P* < 0.001, two-tailed unpaired Student’s *t*-test.


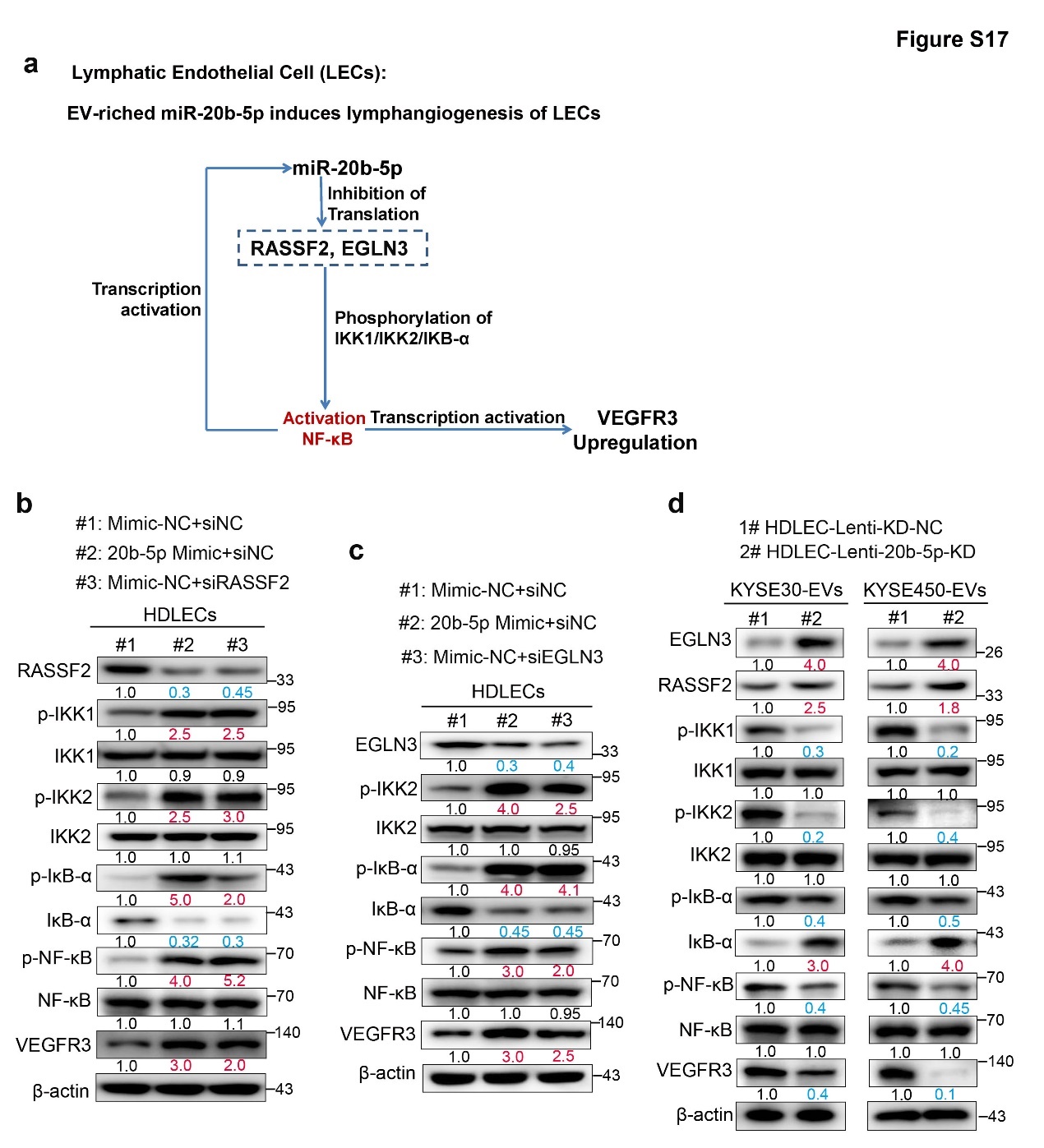


**Figure S17. EV miR-20b-5p transactivates VEGFR3 by targeting RASSF2/EGLN3-NF-κB signaling pathway.**

1. Schematic model of the mechanism of EVs carrying miR-20b-5p in HDLECs.
2. Western blot analysis of the indicated proteins in HDLECs transfected with the miR-20b-5p mimic or RASSF2 siRNA.
3. Western blot analysis of the indicated proteins in HDLECs transfected with the miR-20b-5p mimic or EGLN3 siRNA.
4. Western blot analysis of the indicated proteins in HDLEC-Lenti-miR-20b-5p-KD or HDLEC-Lenti-KD-NC cultured with EVs from KYSE30/KYSE450.


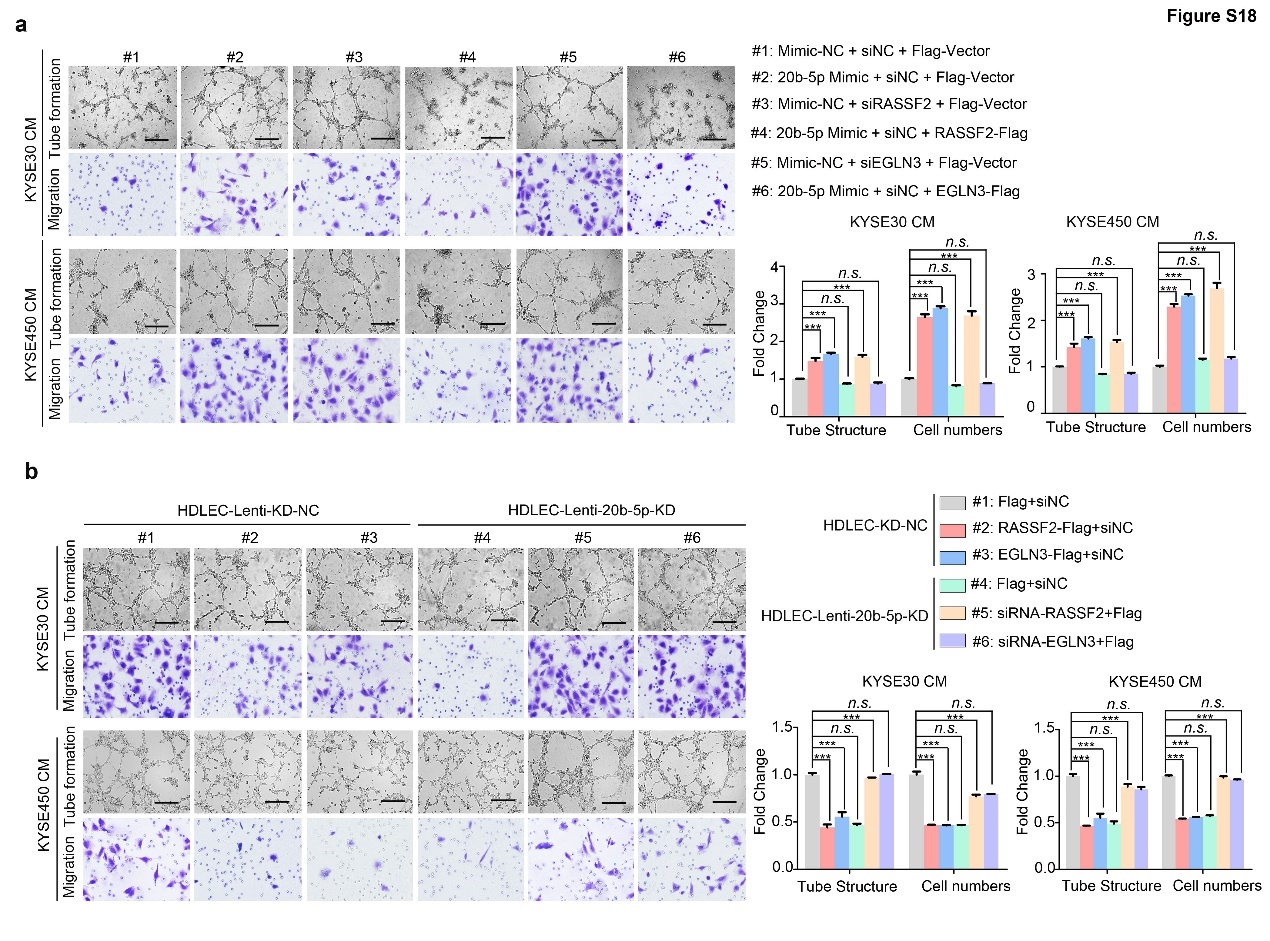


**Figure S18. RASSF2 and EGLN3 are responsible for the EV-riched miR-20b-5p effect on HDLEC** **migration and tube formation.**

**(a)** Representative images of HDLECs transfected with the miR-20b-5p mimic or RASSF2/EGLN3 siRNA or cotransfected with the miR-20b-5p mimic and the RASSF2/EGLN3 plasmid; the cells were cultured with KYSE30/KYSE450 CM. Matrigel tube formation assay (Scale bar, 2mm) and migration assay (Original magnification, ×100).

**(b)** Representative images of HDLECs stably knocked down miR-20b-5p (Lenti-miR-20b-5p-KD) and transfected with RASSF2/EGLN3 siRNA and HDLEC-Lent-KDi-NC cells transfected with the RASSF2/EGLN3 plasmid; the cells were cultured with KYSE30/KYSE450 CM. Matrigel tube formation assay (Scale bar, 2mm) and migration assay (Original magnification, ×100).

The data are representative of three independent experiments. The error bars represent the SEM. ****P* < 0.001; two-tailed unpaired Student’s t-test.


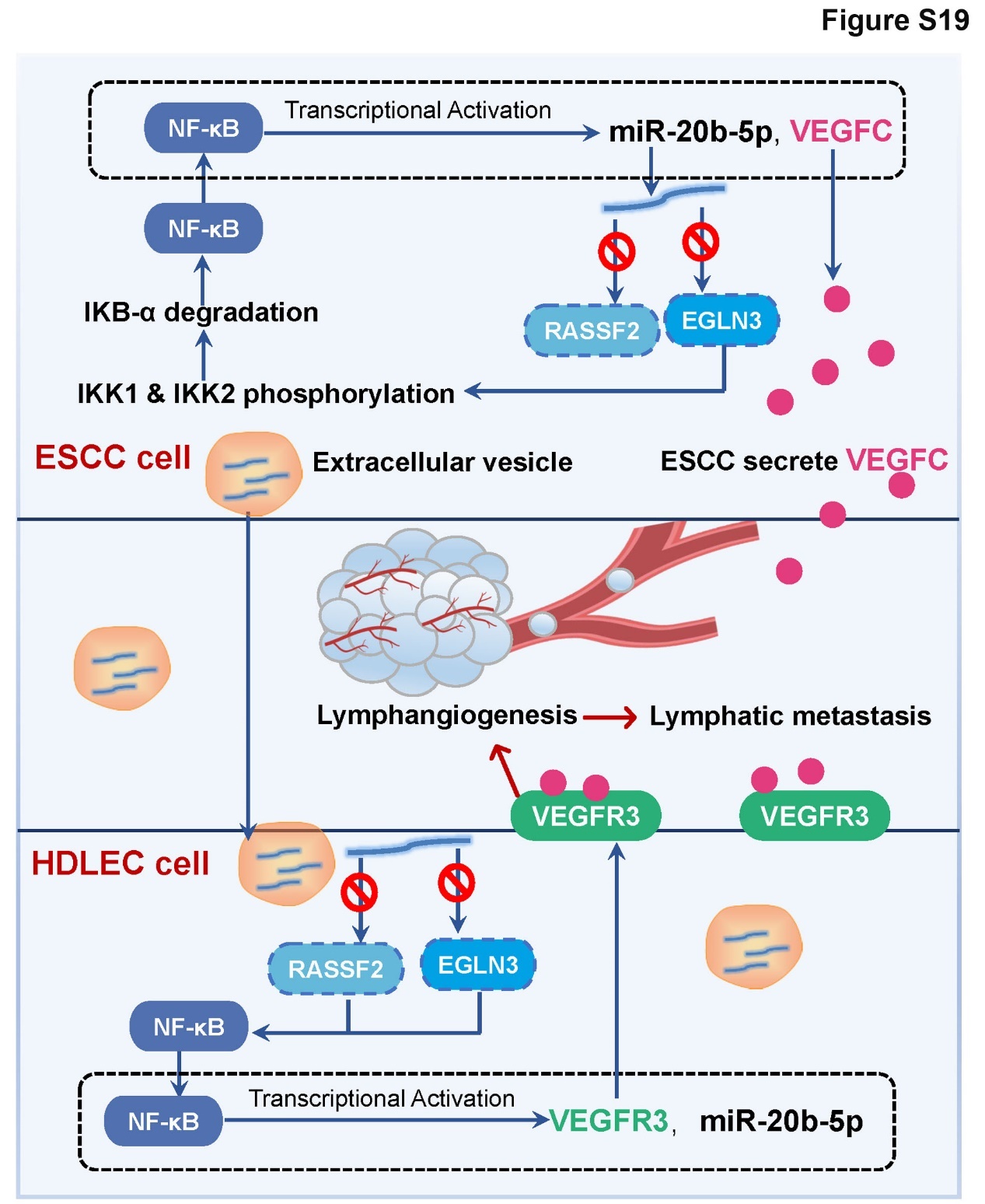


**Figure S19.** **Model of EV miR-20b-5p remodelling the tumour microenvironment by inducing lymphangiogenesis of LECs in ESCC LNM.**

The Figure was created by ScienceSlides Software, Microsoft Office PowerPoint Software and Figdraw.

**Supplementary Table S1. Primers used in this study.**

| hsa-miR-20b-RT primer | GTCGTATCCAGTGCAGGGTCCGAGGTATTCGCACTGGATACGACctacct |
| --- | --- |
| hsa-miR-20b-AS primer | CGCCAAAGTGCTCATAGTGCA |
| common reverse primer | GTGCAGGGTCCGAGGT |
| U6-forward primer | CTCGCTTCGGCAGCACA |
| U6-reverse primer | AACGCTTCACGAATTTGCGT |
| β-actin-forward primer | GAGAAAATCTGGCACCACACC |
| β-actin-reverse primer | GGATAGCACAGCCTGGATAGCAA |
| GAPDH-forward primer | TATGACAACAGCCTCAAGAT |
| GAPDH-reverse primer | AGTCCTTCCACGATACCA |
| Gm13008-forward primer | GCTTTGTGGCCATTGTGCAT |
| Gm13008-reverse primer | CATTTGCCCGTCCCAATGTC |
| hsa-miR-16-RT primer | GTCGTATCCAGTGCAGGGTCCGAGGTATTCGCACTGGATACGACcgccaa |
| hsa-miR-16-AS primer | gAGCAGCACGgAAAgAggG |
| RASSF2-forward primer | CTCTGAAGCCCCTGACTGTG |
| RASSF2-reverse primer | AGTCTGTGGAGCTTGGCATC |
| EGLN3-forward primer | AGTACATCGTGCCCTGTCTG |
| EGLN3-reverse primer | GTGTCGCTTGGAGACGCC |
| NF-κB-CHIP-primer 1-forward primer | GCGCGTTTTTGGTGCTTTG |
| NF-κB-CHIP-primer 1-reverse primer | CGCCGCCGAAATTAAAGAGA |
| NF-κB-CHIP-primer 2-forward primer | ATTTAGTGGCATAAGATATCGTGAC |
| NF-κB-CHIP-primer 2-reverse primer | TGTAATACGTTAAACGAGTGAGGGA |
| NF-κB-CHIP-primer 3-forward primer | TTTCTATTAATGGGGTGGGGGC |
| NF-κB-CHIP-primer 3-reverse primer | GAGGACCTAGAGAAAGGAGCA |
| NF-κB-CHIP-primer 4-forward primer | CAGGGTGGATAGAATGTGCCT |
| NF-κB-CHIP-primer 4-reverse primer | ACACTGGCAACTGCAAAAAGT |
| NF-κB-CHIP-primer 5-forward primer | GATTTGGTTGAAACCACAAAACCT |
| NF-κB-CHIP-primer 5-reverse primer | TAATATTCCTGAAACAGGCTTTCCT |

**Supplementary Table S2. Summary of clinicopathological characteristics of the 106 esophageal squamous cell carcinoma patients (all of them were cM0) with esophagectomy for miR-20b-5p expression detection in the serum and formalin-fixed and paraffin-embedded tumor samples**

| Characteristics | Total  n | pN1-3  n (%) | pN0  n (%) | Lymph node metastasis | |
| --- | --- | --- | --- | --- | --- |
|  |  |  |  | OR (95%CI) | *P* |
| Sex |  |  |  |  |  |
| Male | 86 | 44(51.2) | 42(48.8) | 1 |  |
| Female | 20 | 4 (20.0) | 16(80.0) | 0.239(0.074-0.772) | 0.017 |
| Age |  |  |  |  |  |
| ≤62 | 58 | 28(48.3) | 30(51.7) | 1 |  |
| >62 | 48 | 20(41.7) | 28(58.3) | 0.765(0.354-1.655) | 0.496 |
| Degree of differentiation |  |  |  |  |  |
| Well | 34 | 12(35.3) | 22(64.7) | 1 |  |
| Moderate | 49 | 26(53.1) | 23(46.9) | 2.072(0.843-5.096) | 0.112 |
| Poor | 23 | 10(43.5) | 13(56.5) | 1.410(0.477-4.168) | 0.534 |
| pT category |  |  |  |  |  |
| T2 | 13 | 7(53.8) | 6(46.2) | 1 |  |
| T3 | 83 | 36(43.4) | 47(56.6) | 0.657(0.203-2.123) | 0.482 |
| T4a | 10 | 5(50.0) | 5(50.0) | 0.857(0.164-4.467) | 0.855 |
| miR-20b in serum#（Mean±s.d） | 0.043±0.054 | 0.062±0.070 | 0.028±0.030 | 1.368×10^8^(238.039-7.861×10^13^) | 0.006 |
| miR-20b in FFPE tissue#（Mean±s.d） | 0.435±0.710 | 0.593±0.886 | 0.304±0.494 | 1.841(1.010-3.355) | 0.046 |
| RASSF2 in FFPE tissue# Mean±s.d | 0.053±0.499 | 0.037±0.041 | 0.061±0.059 | <0.001(<0.001-0.301) | 0.025 |
| EGLN3 in FFPE tissue# Mean±s.d | 0.019±0.025 | 0.012±0.019 | 0.025±0.027 | <0.001(<0.001-0.003) | 0.016 |

NA, not associated; FFPE, formalin fixation and paraffin embedding; OR, odds ratio (univariate logistic regression); CI, confidence interval

#As continuous variable

**Supplementary Table S3. Gene symbols of differential gene enrichment analysis in KYSE150-Lenti-OV-NC and KYSE150-Lenti-miR-20b-5p-OV.**

| **Category** | **Term** | **gene symbols** |
| --- | --- | --- |
| **KEGG_PATHWAY** | **hsa04668:TNF signaling pathway** | **PTSG2, LIF, TNFAIP3, PIK3R2, TRAF1, CCL5, IL6** |
| **KEGG_PATHWAY** | **hsa04010:MAPK signaling pathway** | **FGF11, TGFBR1, DUSP5, JUND, FGFR3, NTF4, PPM1A, DUSP7, RELB, NR4A1,NGF** |
| **GOTERM_BP_DIRECT** | **GO:0007250~activation of NF-kappaB-inducing kinase activity** | **TRAF4, ZFP91, TNFSF15** |
| **KEGG_PATHWAY** | **hsa04064:NF-kappa B signaling pathway** | **PTGS2, TNFAIP3, TICAM2, RELB, TRAF1** |
| **KEGG_PATHWAY** | **hsa04151:PI3K-Akt signaling pathway** | **FGF11,FGFR3,PIK3R2,MTCP1, YWHAB,BCL2L11, IL6, CCNE1, NR4A1, NGF,PDPK1** |
| **KEGG_PATHWAY** | **hsa04621:NOD-like receptor signaling pathway** | **CARD6, TNFAIP3, CCL5, IL6** |
| **GOTERM_BP_DIRECT** | **GO:0051726~regulation of cell cycle** | **HIPK2, JUND, CDK19, CCNE1, CENPF, E2F2** |
| **GOTERM_BP_DIRECT** | **GO:0006954~inflammatory response** | **HRH1, PTGS2, IL24, TNFAIP3,TICAM2, SMPDL3B, RELB, CCL5, IL6, AOX1, NDST1, C3** |
| **KEGG_PATHWAY** | **hsa03320:PPAR signaling pathway** | **ACSL5, ACADM, CPT1A, PDPK1** |
| **GOTERM_BP_DIRECT** | **GO:0071456~cellular response to hypoxia** | **HIPK2, PMAIP1, PTGS2, CPEB2, ANKRD1** |

**Supplementary Table S4. Source file of differential gene enrichment analysis in HDLEC-Lenti-miR-20b-5p-KD and HDLEC-Lenti-KD-NC and cultured with CM (including EVs) from KYSE30.**

The Gene Ontology annotation R package Goseq was used in this project. (https://bioconductor.org/packages/devel/bioc/vignettes/goseq/inst/doc/goseq.pdf). The output of this method only contained parameter "numDEInCat", but it did not contain the specific enriched genes in this category. Therefore, we provided the input files, output files and codes of this method to verify the reliability of figures.
